# CRISPR Interference Modules as Low-Burden Logic Inverters in Synthetic Circuits

**DOI:** 10.3389/fbioe.2021.743950

**Published:** 2022-01-28

**Authors:** Massimo Bellato, Angelica Frusteri Chiacchiera, Elia Salibi, Michela Casanova, Davide De Marchi, Ignazio Castagliuolo, Maria Gabriella Cusella De Angelis, Paolo Magni, Lorenzo Pasotti

**Affiliations:** ^1^ Department of Electrical, Computer and Biomedical Engineering, University of Pavia, Pavia, Italy; ^2^ Centre for Health Technologies, University of Pavia, Pavia, Italy; ^3^ Department of Information Engineering, University of Padua, Padua, Italy; ^4^ Department of Molecular Medicine, University of Padua, Padua, Italy; ^5^ Department of Public Health, Experimental and Forensic Medicine, University of Pavia, Pavia, Italy

**Keywords:** CRISPR interference (CRISPRi), cell load, escherichia coli, synthetic circuits, NOT gate

## Abstract

CRISPR and CRISPRi systems have revolutionized our biological engineering capabilities by enabling the editing and regulation of virtually any gene, via customization of single guide RNA (sgRNA) sequences. CRISPRi modules can work as programmable logic inverters, in which the dCas9-sgRNA complex represses a target transcriptional unit. They have been successfully used in bacterial synthetic biology to engineer information processing tasks, as an alternative to the traditionally adopted transcriptional regulators. In this work, we investigated and modulated the transfer function of several model systems with specific focus on the cell load caused by the CRISPRi logic inverters. First, an optimal expression cassette for dCas9 was rationally designed to meet the low-burden high-repression trade-off. Then, a circuit collection was studied at varying levels of dCas9 and sgRNAs targeting three different promoters from the popular *tet*, *lac* and *lux* systems, placed at different DNA copy numbers. The CRISPRi NOT gates showed low-burden properties that were exploited to fix a high resource-consuming circuit previously exhibiting a non-functional input-output characteristic, and were also adopted to upgrade a transcriptional regulator-based NOT gate into a 2-input NOR gate. The obtained data demonstrate that CRISPRi-based modules can effectively act as low-burden components in different synthetic circuits for information processing.

## Introduction

The rational design of new biological functions requires toolkits of components for the engineering of the desired host organisms, as well as circuit composition rules to guarantee a predictable behavior upon interconnection of parts (Schwille, 2011; Cheng and Lu, 2012; Nielsen et al., 2016). Based on the sensing-logic-actuation layering of synthetic circuits, such functions can be implemented to reach the typical system design complexity in the engineering world (Moon et al., 2012). Complexity can be properly handled by decoupling the application-specific sensing and actuator layers, providing interactions with the surrounding environment, from the logic layer, which enables the engineering of complex cellular functions (Wang et al., 2013). Transcriptional regulators are widely used to construct synthetic circuits with increasing complexity, to provide different logic modules that can expand the information processing capabilities of engineered cells (Santos-Moreno and Schaerli, 2020). However, the predictable function of synthetic circuits has been reported to be affected by several factors, like biological noise, cell burden, retroactivity, crosstalk among components and growth environment, limiting the actual complexity that can be reached (Del Vecchio et al., 2008; Arkin, 2013; Xiang et al., 2018; Aoki et al., 2019; Bartoli et al., 2020). Another major issue is the limited availability of toolkits of orthogonal components, restricting the scalability of circuit architectures and also the engineering of non-model organisms. The intrinsic modularity of transcription activator-like effectors (TALEs), zinc finger transcription factors (ZF TFs), and the CRISPR system with the catalytically inactive dead-Cas9 (CRISPR/dCas9 or CRISPR interference—CRISPRi) have been proposed to address such issues (Strauβ and Lahaye, 2013). A major advantage of CRISPRi modules, compared with the traditionally adopted transcriptional regulator proteins, is the easy programmability of sgRNAs to repress the expression of any gene of interest, given the presence of a protospacer adjacent motif (PAM) required for system function (Bikard et al., 2013; Qi et al., 2013). TALEs and ZFTFs are also characterized by a modular structure, but their overall designability remains inferior (Santos-Moreno and Schaerli, 2020). Despite the existence of CRISPR system variants that work at different regulatory levels, the CRISPRi systems operate as transcriptional repressors, easily enabling the construction of logic inverters and NOR gates (Nielsen and Voigt, 2014). CRISPRi modules have already been adopted for the construction of logic gates as parts of interconnected synthetic circuits in bacteria (Nielsen and Voigt, 2014; Mimee et al., 2015; Didovyk et al., 2016; Ceroni et al., 2018; Santos-Moreno et al., 2020; Taketani et al., 2020) and other organisms (Liu et al., 2014; Gao et al., 2016; Gander et al., 2017).

The functioning of complex, but even simple, synthetic circuits can be affected by the unnatural load caused by heterologous gene expression. In bacteria, this load is mainly caused by the limitation of translational resources (Ceroni et al., 2015; Gyorgy et al., 2015) which may decrease upon expression of multiple transcriptional regulators-encoding genes, or even a single one (Carbonell-Ballestero et al., 2016; Pasotti et al., 2017; Qian et al., 2017; Shopera et al., 2017). In the CRISPRi case, translational resources are expected not to be depleted as sgRNAs are only transcribed and the only actors that undergo translation are dCas9 and the target genes. Although the cellular resources that must be dedicated to dCas9 expression can sometimes be significant (Zhang and Voigt, 2018), the functioning of CRISPRi systems as programmable repressors is expected to be less affected by the cell load caused by the different expression levels of sgRNAs during circuit operation.

The architecture of an ideal CRISPRi-based NOT gate includes an input-driven sgRNA expression cassette and a constitutive cassette driving dCas9 at levels that guarantee low burden and toxicity, but a high repression efficiency. In this architecture, the dCas9 cassette can be designed once to meet the requirements above, and the sgRNA sequence customized to target the desired genes without unpredictably affecting ribosome availability for different input levels. The knowledge of the cell load properties of CRISPRi modules is highly relevant to the rational design of biological systems that are expected to show improved predictability in a low-burden setting. However, an in-depth investigation of the cell load caused by sgRNA and dCas9 expression in CRISPRi-based synthetic circuits has not been reported yet. In this work, we aim to demonstrate the low-burden feature of CRISPRi NOT gates by testing a number of model systems in which sgRNAs and dCas9 are tuned over a wide range of transcriptional levels, also showing that cell load in synthetic circuits can be overcome by replacing traditional transcriptional repressors with CRISPRi modules. Issues and relative counteracting methods have been also reported on CRISPRi functioning, namely sgRNA specificity (Didovyk et al., 2016), sgRNA- and dCas9-dependent toxicity (Cui et al., 2018; Zhang and Voigt, 2018) and dCas9-resource limitation (Huang et al., 2021), but such constraints will not be addressed in the present study, which is focused on evaluating the mitigation of the resource limitation issue at the host-circuit interface.

Here, we first constructed a minimal-load dCas9 expression cassette that is able to meet the low-burden high-repression trade-off. Second, we constructed and characterized CRISPRi-based NOT gates in several contexts by engineering customized sgRNAs repressing popular target promoters (i.e., of the *lux*, *lac* and *tet* systems), to understand their efficiency and burden. Finally, CRISPRi-based logic inverters were used as low-burden modules to fix a previously non-working synthetic circuit cascade of transcriptional regulators, and to easily upgrade the input processing capability of an existing transcriptional regulator-based NOT gate by converting it into a 2-input NOR gate.

## Materials and Methods

### Strains, Plasmids and Media

A list of the strains used in this study is reported in Supplementary Table S1, together with a description of all the plasmids, which are available as entries in the MIT Registry of Standard Biological Parts^1^. The pdCas9-bacteria and pgRNA-bacteria were gifts from Stanley Qi (Addgene plasmids #44249 and #44251) (Qi et al., 2013). The *E. coli* TOP10 (Invitrogen) strain was used as a host for cloning and characterization. The strain was transformed by heat shock according to manufacturer’s instructions. L-broth (NaCl 10 g/L, tryptone 10 g/L, yeast extract 5 g/L) was used in plasmid propagation. Antibiotics were added to maintain plasmids in recombinant strains: ampicillin (100 mg/L), kanamycin (25 mg/L) or chloramphenicol (12.5 mg/L), as required by the high-, medium- and low-copy vector backbones pSB1A2, pSB3K3 and pSB4C5, respectively (Shetty et al., 2008). Long-term stocks were made for all the strains by mixing 750 µl of a saturated culture with 250 µl of 80% glycerol, and stored at −80°C. The low-fluorescence M9 supplemented medium (M9 salts 11.28 g/L, thiamine hydrochloride 1 mM, MgSO_4_ 2 mM, CaCl_2_ 0.1 mM, casamino acids 2 g/L and glycerol 0.4%) was used in quantitative assays.

### Cloning

All the plasmids used in this study were constructed through the BioBrick Standard Assembly (Knight, 2003) and conventional molecular biology techniques. As a result, standard DNA junctions (TACTAG upstream of coding sequences, TACTAGAG otherwise) are present between assembled parts. The basic or composite parts used for DNA assembly were retrieved from the MIT Registry 2008–2011 DNA Distribution except for the P_luxRep_ promoter, which was constructed in a previous study (Zucca et al., 2015), the dCas9 gene and sgRNA, which were PCR-amplified from pdCas9-bacteria and pgRNA-bacteria (Qi et al., 2013), and the new parts conceived in this work. The dCas9 gene and sgRNA were PCR-amplified and converted into the BioBrick format to facilitate subsequent DNA assembly steps. DNA assembly involving EcoRI digestion was avoided for dCas9 since two EcoRI restriction sites are present in the coding sequence and were not removed. DNA purification kits (Macherey-Nagel), restriction enzymes and T4 DNA ligase (Roche) and Phusion Hot Start II PCR kit (Thermo Scientific) were used according to manufacturer’s instructions. Plasmids were sequenced *via* the BMR Genomics DNA analysis service (Padova, Italy) and Eurofins Genomics Germany GmbH DNA analysis service (Ebersberg, Germany). Oligonucleotides for mutagenesis were obtained from Metabion International AG (Planegg, Germany) and Eurofins Genomics.

### sgRNA Design and Construction

All the sgRNAs used in this work were designed via the Benchling CRISPR tool^2^, setting a guide length of 20 nucleotides, GCA_00005845.2 as reference genome, and using the Optimized Score by Doench et al., 2016. Mutagenesis with divergent primers was adopted to construct custom sgRNAs and simultaneously delete nucleotides after the transcription start sites of the used promoters, where indicated. Briefly, the template plasmid DNA was purified and used in a mutagenic PCR reaction with the Phusion Hot Start Flex II. The methylated template DNA was digested at 37°C for 1 h with DpnI (Thermo Scientific), directly added at the end of the PCR reaction. The PCR products were run in a 1% agarose gel and then purified. Fifty nanograms of the blunt-ended linear fragments were phosphorylated by polynucleotide kinase (PNK, Thermo Scientific) using the T4 ligase buffer. The reaction was carried out at 37°C for 20 min, then 1 µl of ligase was added and incubated for 16 h at 16°C. The enzymes were deactivated at 75°C for 10 min, the ligation product was transformed, and the mutagenized plasmid was screened via sequencing.

### Fluorescence Assays for Synthetic Circuits Characterization

Fluorescence and absorbance of recombinant strains were measured over time in a microplate reader as previously described (Pasotti et al., 2017). Briefly, bacteria from a glycerol stock were streaked on a selective LB agar plate. After an overnight incubation at 37°C, 1 ml of selective M9 supplemented medium was inoculated with a single colony. Isopropyl-*β*-d-1-thiogalactopyranoside (IPTG, #I1284, Sigma Aldrich) was added to this culture at the desired concentration to initiate the long-dynamics IPTG-dependent gene expression. After a 21 h incubation at 37°C 220 rpm in an orbital shaker, cultures were 100-fold diluted in 200 µl in a 96-well microplate. Two microliters of N-3-oxohexanoyl-l-homoserine lactone (HSL, #K3007, Sigma Aldrich) and/or IPTG were added when required to reach the desired final inducers concentration, in the 0.1–500 nM and 0.1–100 µM ranges, respectively. Cultures were not placed in the external wells of the plate to avoid intensive evaporation. The microplate was incubated with lid in the Infinite F200Pro reader (Tecan) and was assayed via a kinetic cycle: 5 s linear shaking (3 mm amplitude), 5 s wait, absorbance (600 nm) measurement, fluorescence measurements (gain 50 or 80), 5 min sampling time. Red and green fluorescence signals by RFP and GFP were measured with the 535/620 and 485/540 nm filter pairs, respectively. Control wells were also included, as described below, to measure the background signals of absorbance and fluorescence, and to provide internal control references for relative activity calculations. At least three biological replicates, starting from different colonies, were assayed for each strain.

### Data Processing

Data analysis and graphs were carried out *via* GraphPad Prism 8.0.1, Microsoft Excel and Matlab R2017b (MathWorks, Natick, MA). Raw absorbance and fluorescence time series acquired from microplate experiments were background-subtracted, as described in Supplementary Text 1.1. The Matlab *regress* function was used for linear regression fitting in growth rate (µ) calculation. The fluorescence outputs of recombinant strains from microplate experiments were computed in terms of steady-state RFP and GFP synthesis rates per cell (*S*_*cell,RFP*_ and *S*_*cell,GFP*_, in arbitrary units per cell per time unit), expressing the output of synthetic circuits and the cellular capacity indicating the load of the circuit (Ceroni et al., 2015). The average outputs in the exponential growth phase were computed as Eqs 1–3:

$$S\left(t\right)=\frac{dF\left(t\right)}{dt}\cdot \frac{1}{OD_{600}}$$ (1)

$$S_{ave}=mean\left(S\left(t\right)\right)\;\;for\;\;\forall t\in exponential\;growth\;phase\;$$ (2)

$$S_{cell}=\;\frac{S_{ave}}{S_{ave,ref}}\;$$ (3)

where *F(t)* is the background-subtracted fluorescence time series of RFP or GFP, S_ave,ref_ is the S_ave_ of a reference strain (J101R and J101G for RFP and GFP, respectively, see Supplementary Table S1), and the numerical time derivative was used to compute *S(t)*.

### Microscopy

Cultures in M9 supplemented medium, inoculated by single colonies, were incubated at 37°C, 220 rpm overnight and then 20 µl were heat fixed on a glass slide using a Bunsen burner. Fixed cells were stained for 90 s with Gram’s safranin solution (Sigma Aldrich). The staining solution was removed by washing with running tap water and left 10 min to dry under a fume hood. The slide was covered with a drop of immersion oil for microscopy (BM Medical snc, Padua) right before the analysis. A Leica DMLB bright field microscope was used to take pictures of bacterial cells for morphological analysis. Bacteria were magnified with the ×100/1.25 oil immersion objective, and pictures were taken using a Leica DFC7000T digital camera module and processed via Leica Application Suite X. Cell length was quantified via ImageJ (Schneider et al., 2012) and statistics were computed considering 30 cells for each image, evenly sampled from different locations of the acquired pictures.

### Plasmid Copy Number Quantification

The plasmid copy number in recombinant strains bearing two (low- and medium-copy) and three (low-, medium- and high-copy) plasmids was measured *via* gel electrophoresis and image analysis of plasmid DNA from cultures grown overnight in M9 supplemented media, inoculated with a single colony of engineered strain. The low-copy plasmid was assumed to be replicated stably in all the conditions at an average per-cell copy number of 5 (Lee et al., 2011). The medium- and high-copy plasmids were quantified through fluorescence measurements of their DNA fragments upon plasmid purification (Macherey-Nagel Plasmid Kit), restriction digests, and 1% agarose gel electrophoresis stained with ethidium bromide, assuming that the relative amount of all the plasmids does not change during extraction from bacterial cultures. Gel pictures were taken with an Imager CHEMI Premium (VWR) and the fluorescence intensity of bands was analyzed via ImageJ. The GeneRuler 1 Kb DNA ladder was used to assess the linearity of fluorescence intensity of bands as a function of their length, according to the DNA amount, available from the manufacturer, for each band.

### Mathematical Modelling

The interplay among dCas9, sgRNA and target promoter DNA shapes the output of the logic inverter circuits that was simulated by the following biomolecular reaction system Eq. 4.

$$\{\begin{array}{c} C+g\;\begin{array}{c} k1_{+}\\ ⇄\\ k1_{-} \end{array}\;C:g\\ C:g+D\;\begin{array}{c} k2_{+}\\ ⇄\\ k2_{-} \end{array}\;C:g:D \end{array}$$ (4)

where *C*, *g* and *D* represent the intracellular concentrations (nM) of dCas9, sgRNA and free promoter DNA, *C:g* represents the repressor complex and *C:g:D* the repressed target promoter DNA. By naming *C*_*tot*_, *g*_*tot*_ and *D*_*tot*_ the total levels of *C*, *g* and *D,* conservation laws can be defined Eq. 5.

$$\{\begin{array}{c} C_{tot}=C+C:g+C:g:D\\ g_{tot}=g+C:g+C:g:D\\ D_{tot}=D+C:g:D \end{array}$$ (5)

Dissociation constants, expressed in nM concentrations, can also be defined Eq. 6.

$$\{\begin{array}{c} K_{1}=\frac{k1_{-}}{k1_{+}}\\ K_{2}=\frac{k2_{-}}{k2_{+}} \end{array}$$ (6)

Assuming that transcription, translation and molecules degradation and dilution are much slower than the binding rates involved in Eq. 4, the intracellular level of the molecules of interest can be derived using the law of mass action and steady-state hypothesis to obtain an implicit equation system Eq. 7 that was solved via Matlab R2017b (MathWorks) using the fixed-point method as previously reported (Pasotti et al., 2017).

$$\{\begin{array}{c} C=\frac{C_{tot}}{1+\frac{g}{K_{1}}+\frac{g\cdot D}{K_{1}\cdot K_{2}}}\\ g=\frac{g_{tot}}{1+\frac{C}{K_{1}}+\frac{C\cdot D}{K_{1}\cdot K_{2}}}\\ D=\frac{D_{tot}}{1+\frac{g\cdot C}{K_{1}\cdot K_{2}}}\\ R=\theta \cdot D \end{array}$$ (7)

The free promoter DNA (*D*) is assumed to be proportional to the per-cell RFP output (*R*) of the circuits, using the lumped parameter *θ* that includes transcription, translation, fluorophore maturation, molecule degradation and dilution processes, and links the unbound promoter DNA with RFP under the assumption that the target promoter has no basic transcriptional activity in the repressed state. For the sake of simplicity, protein maturation and the constitutively-expressed transcriptional regulators were neglected in this analysis. The proportion of free and total promoter DNA (*D*/*D*_*tot*_) has been used to visualize the shape of the *g*_*tot*_-*D* and *C*_*tot*_-*D* transfer functions. Under the *g* >> *C* >> *D* assumption, an explicit expression of *R* can be obtained Eq. 8 and was occasionally used when indicated.

$$R=\frac{\theta \cdot D_{tot}}{1+\frac{C_{tot}/K_{2}}{1+\frac{1}{g_{tot}/K_{1}}}}\;$$ (8)

Parametrization was carried out using biologically-plausible dissociation constants values of 0.3 and 2 nM for *K*_*1*_ and *K*_*2*_, respectively (Josephs et al., 2015; Wright et al., 2015), DNA concentration values from 1 to 100 nM and dCas9 and sgRNA concentrations from 1 nM to 10 μM, considering that 1 nM approximately corresponds to an intracellular level of about one molecule. The dCas9 and sgRNA concentrations were also decreased for plotting purposes to better visualize the effects over wider ranges of values.

## Results and Discussion

### Circuit Design

**FIGURE 1:**
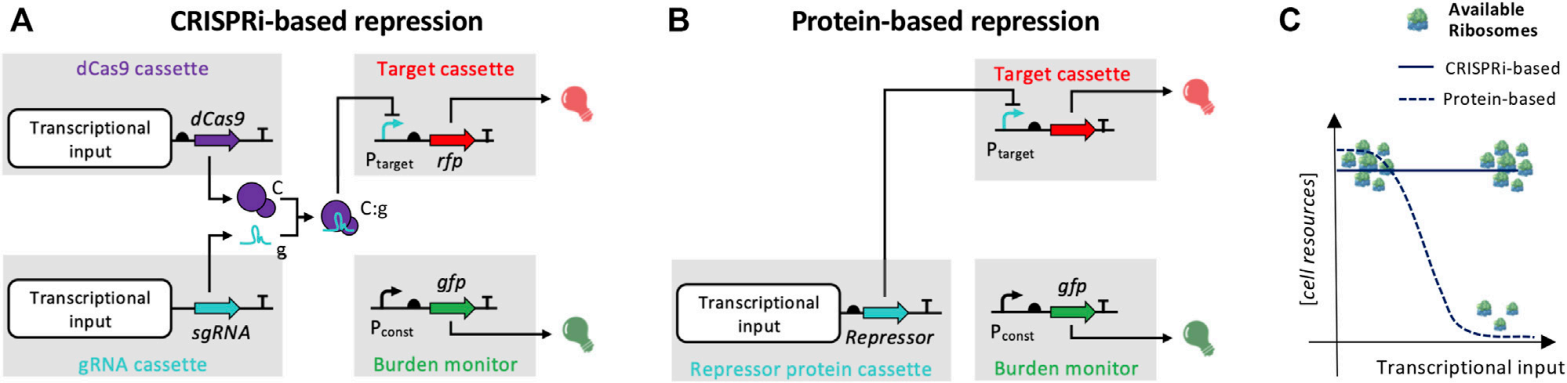
Architecture of the circuit collection used in this work and comparison with traditional protein repressor-based circuits. **(A)** Four modules are illustrated: dCas9, sgRNA, RFP and GFP expression cassettes. Straight arrows represent protein-coding genes or sgRNA, curved arrows represent promoters, half ovals represent ribosome binding sites, and T shapes represent transcriptional terminators. Thin truncated arrows represent repression, while red and green bulbs indicate fluorescent outputs. The *C*, *g* and *C:g* symbols correspond to the dCas9 protein, sgRNA molecule and their complex, respectively, with the same representation as in the mathematical model. Generic transcriptional inputs are shown for the dCas9 and sgRNA cassettes, which may be provided by constitutive or inducible promoters. P_const_ is the J23100 promoter which drives the constitutive expression of GFP in the cell load monitor cassette, and P_target_ is the P_LtetO1_, P_LlacO1_ or the P_luxRep_ promoter. CRISPRi-based logic inverters conform to the illustrated circuitry: they have a tunable transcriptional input driving sgRNA expression, and a constitutive cassette for dCas9 production. **(B)** Architecture of a traditional protein repressor-based logic inverter, shown for comparison: a gene coding for a repressor protein is expressed by a generic transcriptional input and binds a target promoter. Symbols are the same as in panel **(A)**. **(C)** Expected difference between CRISPRi- and protein repressor-based logic inverters in terms of cellular resource usage as a function of the transcriptional input. The expression of repressor protein can exert an input-dependent load for the host, overloading the translational machinery. Conversely, changes of sgRNA expression do not affect ribosome availability, which is expected to be poorly affected, assuming that dCas9 expression is optimized to guarantee a low impact on translational resources during circuit operation.

The architecture of the circuits used in this study is shown in Figure 1. All circuits relied on three gene cassettes for the expression of dCas9, sgRNA and RFP. The promoters used to drive RFP were P_LtetO1_, P_LlacO1_ or P_luxRep_, which have been previously used in many synthetic circuits with their cognate regulators, i.e., from the popular *tet*, *lac* and *lux* systems, respectively, but in this circuit collection they are only regulated by specific sgRNAs. The dCas9 cassette was either inducible (driven by P_lux_) or constitutive (driven by the J23116 promoter from the Anderson collection^1^), with design details specified in the next section. The sgRNA cassette was based on the design by Jinek et al., 2012, and a 20-nt sequence was customized to target the P_LtetO1_, P_LlacO1_ or P_luxRep_ promoters, covering at least one nucleotide of the −35 region. When an inducible dCas9 cassette was present, the sgRNA was driven by a constitutive promoter (J23116, J23100 or J23119, ordered from the weakest to the strongest one). When the dCas9 was constitutive, the sgRNA was expressed by an inducible device (*lux* or *lac* system). Inducible cassettes of dCas9 and sgRNAs were always placed in low-copy vector. The constitutive cassette of dCas9 was placed in medium-copy vector, the sgRNA cassettes were assembled in either low- or medium-copy vector, and the RFP cassette was placed in either medium- or high-copy vector. As a result, two-to three-plasmid engineered strains were constructed, including low/medium, low/high and low/medium/high copy plasmids. The inducible devices driving the expression of dCas9 or sgRNAs were selected not to interfere with the regulation of RFP and required a constitutively expressed transcriptional regulator gene (*luxR* or *lacI*) to enable induction. Finally, a GFP expression cassette was also included in every circuit, assembled in the low-copy vector, as a proxy of cell load (Ceroni et al., 2015; Gyorgy et al., 2015; Pasotti et al., 2017).

According to the activity of the used promoters and the copy numbers of the vectors, the described circuits can span a very wide range of expression levels for dCas9 and sgRNAs, which eventually enables to investigate their effect on the RFP target gene and on cell load.

### Simulations of Circuit Behaviour

**FIGURE 2:**
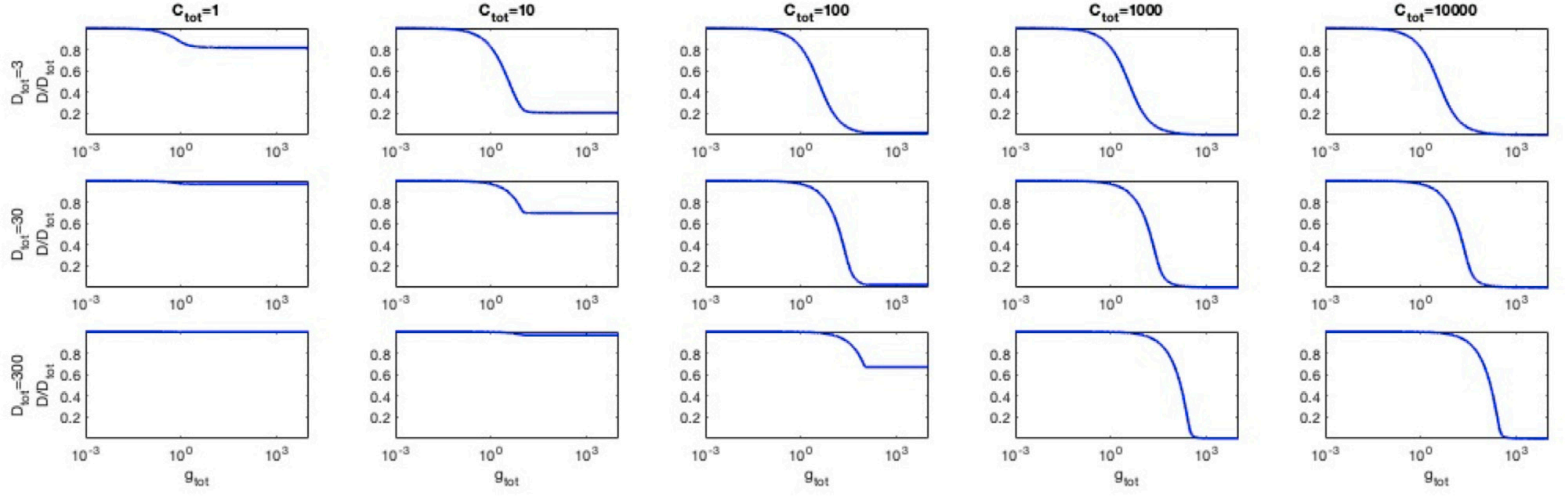
Mathematical model simulations of circuit output as a function of dCas9, sgRNA and target promoter DNA copy number. The output curves represent the intracellular concentration of free promoter DNA (*D*) normalized by the total concentration of available promoter DNA (*D*_*tot*_). The independent variable of the simulations is the intracellular concentration of sgRNA (*g*_*tot*_, expressed as nM concentration). Simulations are shown for different values of dCas9 (*C*_*tot*_, expressed as nM concentration, in the columns) and DNA (*D*_*tot*_, expressed as nM concentration, in the rows). Parameters: K_1_ = 0.3 nM, K_2_ = 2 nM.

Computational modelling was used to simulate the steady-state of RFP output as a function of dCas9, sgRNA and target promoter DNA copy number, to evaluate the expected behavior of the designed circuits. The simulated output curves are reported in Figure 2. They show a complex relationship among dCas9, sgRNA and DNA levels, which affect the output gene nonlinearly. The traditionally used Michaelis-Menten equation models rely on different simplifying assumptions, namely the abundance of sgRNA compared with dCas9, and of dCas9 compared with DNA molecules Eq. 8. However, in this work we aimed to model large fold-changes of each molecule, making such assumptions unsuitable and requiring a more general model Eq. 7. The model of Eq. 7 shows a DNA level-dependent switch point for the repression curve, which increases when DNA copy number increases. This indicates that engineered strains tested with identical dCas9 and sgRNA expression levels can have very different output shapes for different target promoter DNA copy numbers, and that their output may not only change by a scale factor as predicted by the Michaelis-Menten function in Eq. 8. In addition, the model in Eq. 7 shows that saturating amounts of dCas9 may provide incomplete repression when sgRNA is not highly expressed, and the effect is also DNA level-dependent. This behaviour was expected, since a sufficient concentration of the dCas9:sgRNA repressor complex cannot be reached, and the DNA concentration dictates the critical dCas9:sgRNA level to achieve a complete repression of the output. This indicates that engineered strains will show a non-zero RFP expression for high levels of dCas9 when sgRNA expression is not sufficiently high and when DNA copy number is not low enough. As above, the Michaelis-Menten model in Eq. 8 did not describe this behavior, relying on an sgRNA abundance assumption compared with the other intracellular species.

As expected from the structure of Eq. 7, changes of sgRNA or dCas9 concentrations have identical effects in the sgRNA-dCas9-DNA relationship and for this reason the trends in Figure 2 still persist if *g* and *C* are exchanged (Supplementary Figure S1). A variation of the dissociation constant parameters values was simulated, without changing the reached conclusions (Supplementary Figure S2). The intracellular concentrations of the two species are the result of expression and degradation at the level of RNA and, for dCas9, also of the protein. According to the design specifications followed in this work, the expression of sgRNA and dCas9 is tuned using constitutive or inducible promoters, but the translation process of the dCas9 gene and the very different degradation rates of the two molecules make a comparison of the intracellular levels of sgRNA and dCas9 difficult. Therefore, the model recapitulates the expected behaviour of circuits when the intracellular levels of sgRNA and dCas9 become different regardless of the specific tuning mechanism. It has been demonstrated that constitutive or inducible expression of dCas9 and guides have different effects on the biological noise of the target gene, making the choice of such gene expression platform essential to study cell-to-cell variability (Vigouroux et al., 2018), but this feature is beyond the scope of this work. Taken together, the designed circuit collection is expected to provide wide ranges of repression and its tunability, making them suitable for the design of logic inverters.

### Tuning of dCas9 Expression Level

A multi-faceted approach was adopted to find an optimal expression level of dCas9, measuring growth rate and GFP as indicators of cell load (Ceroni et al., 2015), RFP as indicator of repression efficiency on target gene, and cell morphology as indicator of dCas9 toxicity (Cho et al., 2018). We searched for a trade-off to achieve a minimal-burden and maximum repression in case of a single sgRNA, while analogous procedures could be followed in case of multiple guides. Such optimization is key, since an unbalanced expression could cause severe growth defects, and previous efforts were dedicated to search for a similar trade-off based on growth rate vs. repression measures (Nielsen and Voigt, 2014) and on the screening of randomized RBS libraries (Depardieu and Bikard, 2020).

**FIGURE 3:**
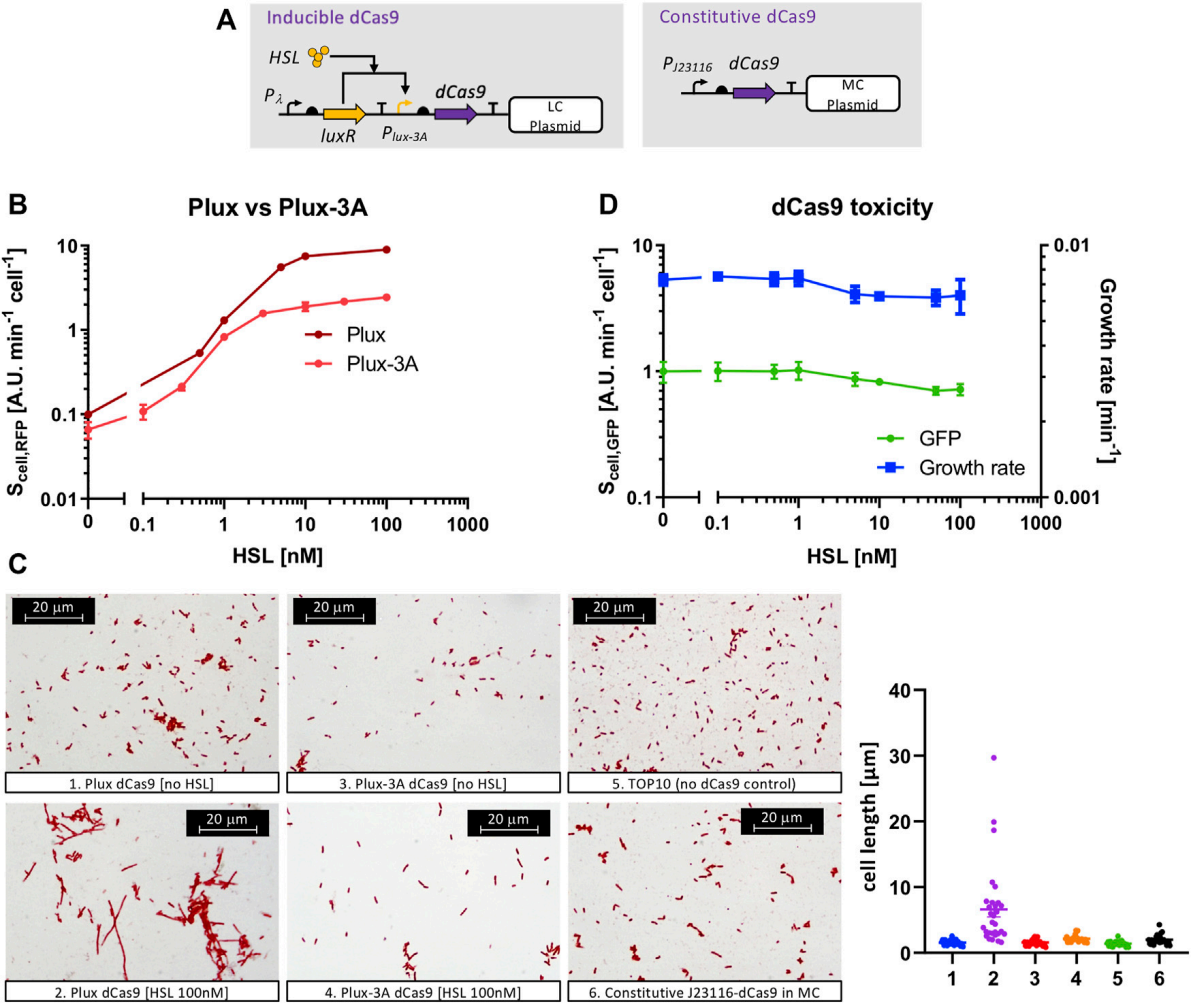
Model systems to study the phenotypic effects of dCas9 in terms of cell load and toxicity. **(A)** Synthetic circuits for the HSL-inducible (left) or constitutive (right) expression of dCas9. Straight arrows represent protein-coding genes or sgRNA, curved arrows represent promoters, half ovals represent ribosome binding sites, T shapes represent transcriptional terminators. Thin truncated arrows represent repression. The HSL-inducible construct also includes a constitutively expressed GFP as cell load monitor, not shown in the scheme. P_λ_ acts as a strong constitutive promoter for LuxR expression; P_J23116_ is a weak constitutive promoter; P_lux-3A_ is similar to the wild-type P_lux_ promoter but without three adenines after the transcription start site. **(B)** Transfer functions, with RFP as output, of the HSL-inducible systems including P_lux_ or P_lux-3A_, as indicated (strains Hr and H_-3_r). Data are shown as the average RFP synthesis rate per cell, as a function of HSL. **(C)** Microscopy images of the indicated recombinant strains to observe morphological changes in cell length related to dCas9-induced toxicity. The graph on the right shows the cell length distribution of 30 sampled cells of each picture. Data points indicate the individual cell length values, thick line indicates the mean value and error bars the standard errors of the mean, **(D)** Growth rate and GFP (expressed as the average GFP synthesis rate per cell), as cell load indexes, for the strain bearing the dCas9 inducible circuit (H_-3_d strain) as a function of HSL. In panels **(B)** and **(D)**, data points represent the average value and error bars represent the standard errors of the mean of at least three independent experiments.

**FIGURE 4:**
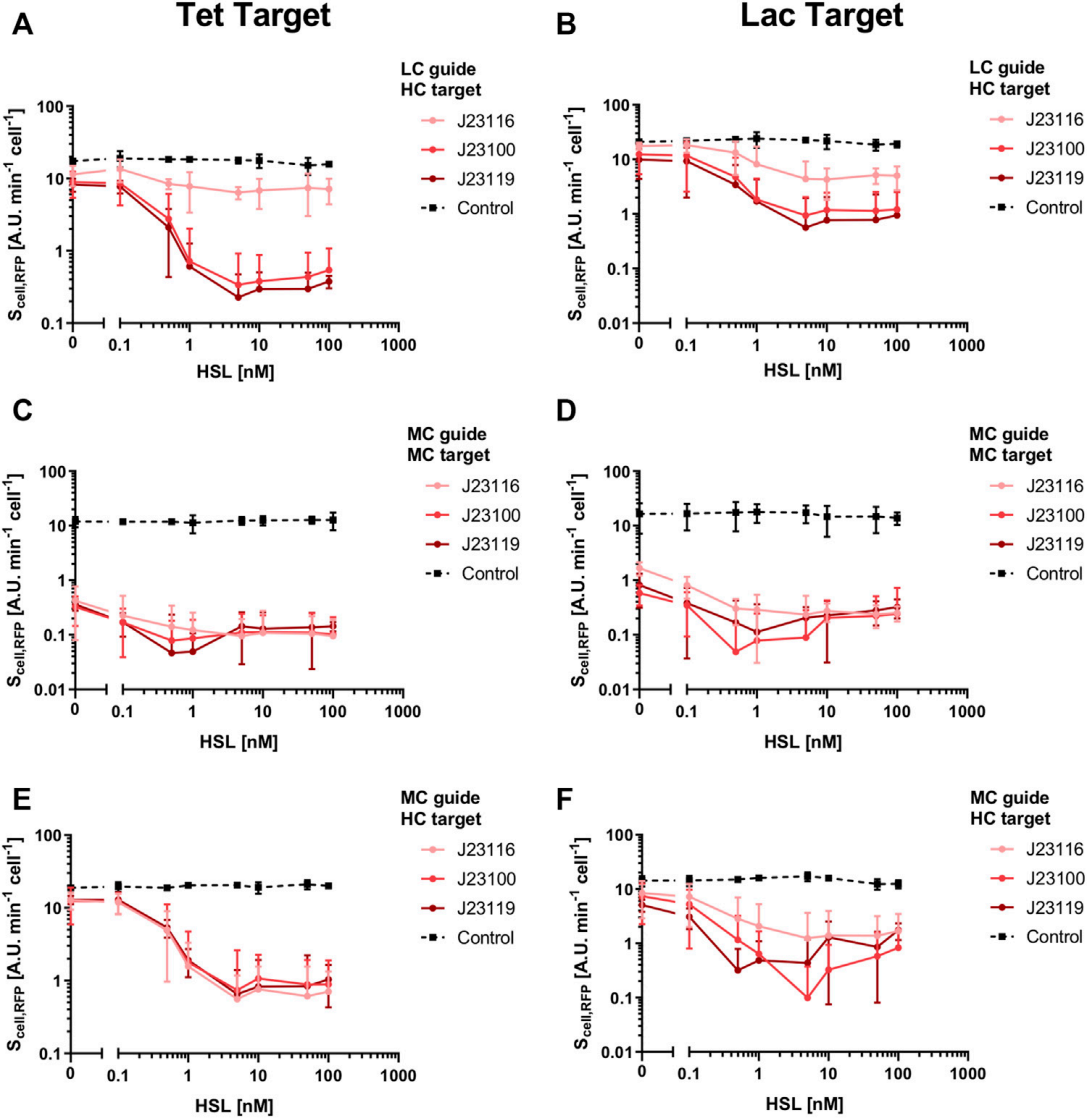
Transfer functions of recombinant strains with HSL-inducible dCas9 and constitutive sgRNA. Transfer curves, with RFP as output, are reported as a function of HSL. Data are shown as the average RFP synthesis rate per cell. In each panel, the copy number of the sgRNA constitutive cassette (low copy—LC, medium copy—MC) and the copy number of the target (medium copy—MC, high copy—HC) are reported. Two different targeting systems [Tet—panels **(A,C,E)**, and Lac—panels **(B,D,F)**] are reported: gPtet and gPlac, which repress the P_LtetO1_ and P_LlacO1_ promoters, respectively, that drive RFP. Each panel includes four curves: three of them correspond to circuits with the sgRNA under the control of three different constitutive promoters of diverse strengths (weak, medium and strong for J23116, J23100 and J23119, respectively), and one curve corresponds to a non-specific targeting control in which the medium-strength J23100 promoter constitutively transcribes a non-targeting sgRNA: gPlac and gPtet for the P_LtetO1_ and P_LlacO1_ promoters in the Tet and Lac systems, respectively. Data points represent the average value and error bars represent the standard errors of the mean of at least three independent experiments.

We used a platform circuit in which dCas9 was driven by either an inducible or a constitutive promoter (Figure 3A). The first attempt included the expression of dCas9 on a low-copy vector under the control of the wild-type P_lux_ promoter (R0062 from the Registry of Standard Biological Parts) and a strong RBS, originally present in the pdcas-bacteria template plasmid. The P_lux_ promoter is able to tune the transcription of the downstream genes over a wide range of levels upon HSL addition, as it was tested by assembling an RFP gene downstream (Figure 3B). However, a filamentous cell morphology was detected for this strain at high inducer level (100 nM of HSL), even though the cell population growth was not inhibited (Figure 3C). The same strain without HSL showed a phenotype similar to the non-engineered TOP10 control (Figure 3C). The filamentous phenotype was a sign of toxicity, previously found by Cho et al., 2018, and indicated that the full range of HSL-dependent transcriptional activities could not be exploited without severe defects. We modified the 5′-UTR of the dCas9 transcript to decrease its translation efficiency by removing the three adenine nucleotides after the transcription start site (TSS) that were originally present in the P_lux_ sequence. This modification (indicated as P_lux-3A_) was predicted to decrease the translation initiation rate (TIR) of dCas9 and also of RFP, according to the RBS Calculator tool (Reis and Salis, 2020). We tested this intervention on an HSL-inducible RFP system (Figure 3B) to demonstrate that the P_lux-3A_ promoter was still fully functional and the output RFP level was about 2.5-fold lower than the original P_lux_ promoter, due to a different TIR. To demonstrate that the observed difference in RFP level is due to translation and that the transcriptional activity of P_lux_ and P_lux-3A_ is not affected, a specific experiment described below (see the *Applications to synthetic circuit design* section and Supplementary Figure S10B) was carried out by investigating the P_lux_- and P_lux-3A_-driven expression of an sgRNA, which is transcribed but not translated. Since this experiment showed no relevant decrease in sgRNA expression with P_lux-3A_, we concluded that the lower RFP level was most probably due to a decrease in translation efficiency in the strain with P_lux-3A_. The removal of the 3A nucleotides in the P_lux_ promoter made the toxicity of the HSL-inducible dCas9 circuit negligible at high inducer level, as shown by the microscopy images in Figure3C. Cell load, related to the resource usage caused by the consumption of cellular resources in heterologous expression instead of protein toxicity, was measured in the same strain in terms of growth rate and GFP (Figure 3D). Both measures well correlate and show a null toxicity up to 1 nM of HSL. At higher inducer levels a slight decrease of cell growth and GFP was observed, even though the reached values were not expected to cause relevant issues in terms of cell load (the growth rate and GFP values at 100 nM of HSL correspond to 86 and 71% of the condition without HSL). These data suggested that this inducible system was able to investigate the effects of dCas9 over a wide range of values without significant cell load or toxicity. To evaluate the repression efficiency and to investigate if an optimal balance between efficiency and load could be found, we tested 18 circuits (with their six controls) with the architecture described in Figure 1. All of them had an HSL-inducible dCas9 in low-copy, a constitutive sgRNA driven by weak, medium or strong promoter in low- or medium-copy, and the RFP target was present in medium- or high-copy. Finally, two sgRNAs called gPtet and gPlac, targeting the P_LtetO1_ and P_LlacO1_ promoters, respectively, were considered. Control strains were included for each target promoter and had a non-specific sgRNA driven by the J23100 medium-strength promoter, i.e., gPtet for P_LlacO1_ and gPlac for P_LtetO1_. The results, reported in Figure 4, showed that a very diverse RFP expression could be achieved. All of the circuits with medium-copy RFP targets are almost fully repressed (about 100-fold compared with the control with non-specific guide) regardless of the sgRNA sequence and expression. This demonstrates that the basic activity of P_lux-3A_ in low-copy and a weakly expressed sgRNA in medium-copy are already capable of efficiently repressing the target promoter. The control strains with non-specific guides were not affected by HSL and exhibited a high RFP expression, as expected. The high-copy target circuits showed a higher tunability and the main features depicted in the model simulations. In specific, although none of them reached the same RFP level of their respective controls, RFP expression was high for HSL concentrations up to 0.1 nM and reached a repressed state for HSL values of 1 nM. The striking difference between copy number conditions of the target was predicted by the model, in which a promoter copy number-dependent repression trend was observed. The weakest expression cassettes for sgRNAs (driven by J23116) failed to achieve a complete RFP repression even for saturating amounts of dCas9, as confirmed in the mathematical analysis. Qualitatively, the same trends were observed for both gPtet and gPlac. The medium-copy gPlac and high-copy RFP condition failed to provide evolutionary stable strains, i.e., a significant number of mutant colonies were visible upon streaking on selective LB agar plates and any strain reconstruction or colony isolation attempt was unable to solve the issue (data not shown). For this reason, the respective data should be considered with caution.

Growth rate and GFP expression data, reported in Supplementary Figure S3 and Supplementary Figure S4, confirmed the slight decrease observed for high dCas9 expression level, and showed no sgRNA expression-dependent burden among the circuit variants. In the high-copy target conditions, GFP showed an expression peak at 1 nM of HSL, corresponding to a situation in which RFP expression is low and dCas9 expression is not toxic. A systematically lower GFP level is observed for the controls compared with their respective circuits in the same conditions with specific guides, most probably due to the higher load caused by RFP expression. Taken together, the results showed that none of the tested conditions exhibited cell load as a function of the sgRNA expression strength, and the repression capability is extremely high for all the circuits, starting from the 1 nM inducer concentration. The 1 nM of HSL optimum found in the GFP data of several circuits confirms that the best balance for our platform occurs for dCas9 expression of about 0.8 AU (Figure 3B). Based on this finding, we constructed a constitutive minimal burden dCas9 expression plasmid in which the J23116 promoter was chosen to drive the dCas9 gene in a medium-copy vector, approaching the desired expression level according to the strength of our in-house collection of promoters in medium-copy (data not shown). Morphological analysis of a strain bearing this constitutive dCas9 plasmid showed no filamentous phenotype (Figure 3C) and was adopted to construct sgRNA-based logic inverters. A quantification of cell length by image analysis confirmed the above conclusions (Figure 3C): an average length of 1.6 µm was observed for both P_lux_- and P_lux-3A_-driven dCas9 expression systems without inducer, with the control strain showing a similar average length (1.4 µm). Induction with 100 nM of HSL in the P_lux_-driven dCas9 expression strain increased the average cell length to 6.6 µm, with a relevant amount of cells showing filaments longer than 10 μm, consistent with the observations reported by Cho et al., 2018, but only a modest increase of cell length was observed for the P_lux-3A_ expression strain (2.2 µm average length). The constitutive dCas9 strain also showed a normal average length (2 µm), confirming the low toxicity in all the conditions except the P_lux_-driven dCas9 upon HSL induction.

### Logic Inverters Characterization

**FIGURE 5:**
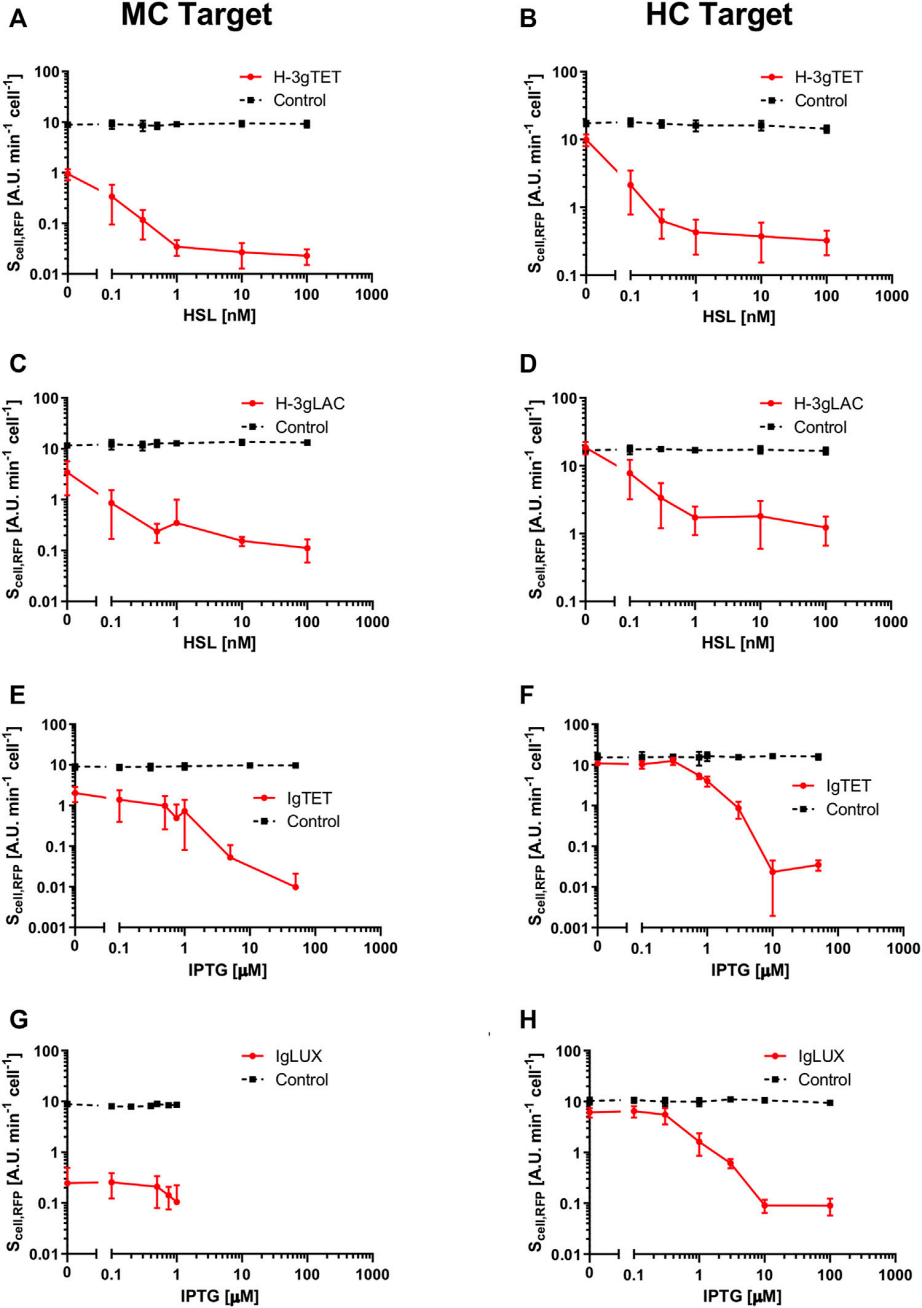
Transfer functions of recombinant strains with constitutive dCas9 and inducible sgRNA. Transfer curves, with RFP as output, are reported as a function of the inducer concentration driving sgRNA expression [HSL, in panels **(A–D)**, or IPTG, in panels **(E–H)**]. Data are shown as the average RFP synthesis rate per cell. In each panel, the CRISPRi targeting system with gPtet, gPlac and gPluxH, which repress the P_LtetO1_, P_LlacO1_ and P_luxRep_ target promoters, respectively, is shown, using the strain nomenclature of Supplementary Table S1. In particular, H_-3_gTET, H_-3_gLAC, IgTET and IgLUX indicate HSL-inducible gPtet, HSL-inducible gPlac, IPTG-inducible gPtet and IPTG-inducible gPluxH cassettes in low-copy plasmid. Two different copy number contexts for the target are reported: medium copy (MC) and high copy (HC). Each panel includes two curves, corresponding to circuits with specific or non-specific targeting system. The latter is referred to as control and the used sgRNAs are gPlac [panels **(A,B)**], gPtet [panels **(C,D)** and **(G,H)** and gPluxH **(E,F)]**. Data points represent the average value and error bars represent the standard errors of the mean of at least three independent experiments.

A set of eight circuits, including a constitutive dCas9 cassette and inducible sgRNA, with their eight respective controls with non-specific guides, were characterized. The design of these model systems resembles the structure of actual logic inverters in which the gene expressed in an input-dependent fashion is the sgRNA. The studied circuits included an input-controlled guide (gPtet, gPlac or gPluxH) targeting the P_LtetO1_, P_LlacO1_ or P_luxRep_ promoter, respectively, that express RFP in medium- or high-copy vector. To design circuits with orthogonal input and output modules, gPlac was expressed by an HSL-inducible module, gPluxH was expressed by an IPTG-inducible module, and gPtet was tested downstream of either HSL- or IPTG-inducible devices. The P_lux-3A_ promoter was used in the HSL-inducible modules since it was found to be functional and it did not include extra-nucleotides in the sgRNA transcript downstream of the TSS. The P_LlacO1_ promoter originally available from the Registry of Standard Biological Parts (R0011) also has an extra adenine after the TSS, but it was not removed in the expression systems shown in this work. Test circuits without this adenine were constructed and they did not show relevant differences (data not shown). Both HSL- and IPTG-inducible devices are characterized by tunable and unimodal behaviour in response to their specific inducer molecules (Zucca et al., 2015; Bandiera et al., 2016). Since the inducible devices driving sgRNAs are different (Figure 3B and Supplementary Figure S5), the transfer functions are inevitably expected to show differences. The obtained input-output data for sgRNA-based logic inverters are reported in Figure 5. All of them showed an efficient repression of RFP when the specific sgRNA is expressed at high levels, demonstrating that the designed constitutive dCas9 cassette is suitable to engineer individual tightly regulated NOT gates. However, their output range was highly dependent on the target copy number, as observed in the inducible dCas9 circuits in the previous section. In fact, at null inducer concentration all the circuits with medium-copy target showed an RFP output lower than 25% of the respective control circuit with non-specific guide, and the gPluxH/P_luxRep_ system even showed an always off state. On the other hand, when the target was moved to a high-copy vector the output was more tunable, with RFP levels from about 60% to 100% of the control. Regarding the unrepressed control circuits, for each promoter the RFP output in high-copy is higher than in medium-copy, as expected. However, the RFP expression difference between medium- and high-copy for all the promoters was less than 2-fold, which is lower than expected from the copy number fold difference (2 to 30-fold, depending on the conditions) reported between pSB3K3 and pSB1A2^1^. Since copy number control could be dependent from strain, temperature, media and presence of multiple plasmids in the same cell (Lee et al., 2011; Massaiu et al., 2015), we have quantified the medium- and high-copy plasmid copy number in the used engineered strains (Supplementary Figure S6). We found a relatively constant copy number in the two- and three-plasmid strains, with per cell copy numbers of 12–16 and 69–70 for the medium- and high-copy vectors, respectively, consistent with literature values. These results indicate that no wide variation in circuit copy numbers occur between the two- and three-plasmid conditions, and the observed RFP values in the controls may be affected by cell load, caused by the target protein expression at increasing copy number values, so that a four- to five-fold higher copy number results into a less than 2-fold increase of RFP expression. The same observations also persist for the inducible dCas9 circuits described in the previous section (Figure 4). Growth rate and GFP data, reported in Supplementary Figure S7 and Supplementary Figure S8, indicate that the main source of cell load is provided by RFP expression. In fact, non-specific control strains exhibit equal or lower values of both measures than the repressible circuits. This can be clearly appreciated in the GFP data (Supplementary Figure S8) in which all the controls showed higher load, and the repressible circuits with the highest RFP value at null induction (P_LtetO1_ and P_LlacO1_) in high-copy condition showed an increasing GFP trend as a function of inducer (HSL or IPTG) concentration. This trend occurs because at low inducer levels RFP expression causes a detectable load but, when gPtet and gPlac increase their level, RFP becomes repressed and a lower RFP expression is beneficial to the strain. In these strains, the average cellular capacity benefit of sgRNA induction, in terms of GFP, was 13%. Considering the control circuits, in which RFP expression is constant, no consistent sign of cell load could be associated with the expression of the specific sgRNAs used, and both growth rate and GFP at the maximum induction tested showed about 85% of their value at null induction. This demonstrates that CRISPRi NOT gates with high repression range, in which sgRNAs are tuned over wide expression levels, could be adopted without causing a relevant load to the cells.

**FIGURE 6:**
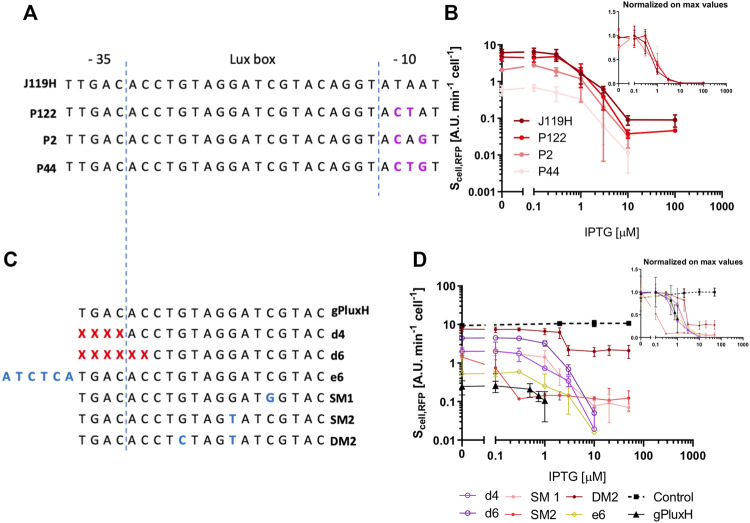
Tunability of CRISPRi modules. **(A)** Architecture of four members of a LuxR-repressible promoter library used in this work. They share the −35 sequence and the lux box in the core region, while the mutations in the −10 sequence affect their transcriptional strengths. The gPluxH guide targets the same region in all the four promoters, from nucleotide 2 to 21. **(B)** Transfer functions, with RFP as output, are reported as a function of IPTG concentration, driving gPluxH expression in four circuits with constitutive dCas9, IPTG-inducible sgRNA, and P_luxRep_, P_122_, P_2_ or P_44_ as target promoter. The inset shows the same graph with values normalized by the maximum data point of each transfer curve. **(C)** Description of the gPluxH variants. Blue nucleotides represent mismatches compared with the target sequence. **(D)** Transfer functions, with RFP as output, are reported as a function of IPTG concentration, driving the gPluxH variants expression in different circuits with constitutive dCas9, IPTG-inducible sgRNA, and P_luxRep_ as target promoter driving RFP in a medium copy plasmid. The control represents an identical circuit but including the gPtet guide, which is not able to target P_luxRep_. In panels **(B,D)**, data are shown as the average RFP synthesis rate per cell and data points represent the average value, with error bars representing the standard errors of the mean of at least three independent experiments.

We then addressed the tunability of the NOT gates transfer functions by modifications of the target promoters or guide sequences to expand our rational engineering capabilities of CRISPRi systems. To investigate the effect of modifications in the target promoter, the availability of a library of promoters sharing several nucleotides with P_luxRep_, previously constructed in our lab (Zucca et al., 2015), was exploited to verify the repression efficiency of the same sgRNA (gPluxH) targeting promoters with different strengths. The P_44_, P_2_ and P_122_ promoters (from the weakest to the strongest), which share the 20-nt gPluxH target sequence with the strongest library member, P_luxRep_ (Figure 6A) were investigated in high-copy conditions, with the IPTG-inducible gPluxH cassette in low-copy plasmid, as in the circuits illustrated above. The resulting data showed that the repression curve as a function of IPTG is very similar among the library members, confirming that the output activity of logic inverters can be tuned by changing promoter sequences from libraries of known strength (Figure 6B). To investigate the effect of modifications in the guide sequences, the underlying assumption was that changes in the 20-nt targeting sequence of the sgRNA are expected to tune the affinity between repressor complex and target DNA, i.e., increase the *K*_*2*_ dissociation constant Eq. 6. This modification could be adopted to improve the output range of the circuits always resulting in the repressed state, i.e., essentially the ones with the target in medium-copy, thus providing another degree of freedom towards the fine tuning of the logic inverters. Deletions (i.e., truncations at the 5’ end), extensions with mismatching nucleotides and mismatches have been previously adopted in other works to modify the traditionally used 20-nt targeting sequence of sgRNAs (Larson et al., 2013; Ceroni et al., 2018; Vigouroux et al., 2018). The gPluxH/P_luxRep_ circuit with the target promoter in medium-copy plasmid was used as a model system to investigate different modifications in gPluxH to obtain a transfer function with high output range. The sgRNA variants and the obtained data are reported in Figures 6C,D and Supplementary Figure S9. Extension achieved up to a 5-fold increase of the RFP value at null induction compared with the circuit with the original gPluxH guide, with the 11-nt extension giving the major increase and the three- and six-nt extensions giving a modest increase. Such increase was still limited since the maximum RFP value was less than 15% of the RFP value of the control, i.e., an identical circuit with a non-targeting guide (gPtet), demonstrating that the P_luxRep_ promoter was still highly repressed. Guides with extensions of more than 11 mismatching nucleotides were not tested. Deletions, tested as truncations of up to five nucleotides, represented a more efficient approach to weaken guide affinity, with the RFP output increasing with the length of the deleted region, up to 20-fold compared with the original gPluxH circuit. This represented about 50% of the output of the control, and the repression efficiency of such guides was still satisfactory, i.e., RFP value was very low at high IPTG concentrations. Finally, the use of mismatched nucleotides in the 20-nt sequence was exploratorily tested with three representative gPluxH variants with one or two mismatches, while an in-depth study is beyond the scope of this work. The single-mismatch guides were not able to provide high output ranges: their RFP value in absence of IPTG did not exceed 20% of the RFP in the control. On the other hand, the double-mismatch gPluxH without IPTG showed more than 75% of the RFP value of the control. However, the IPTG-dependent regulation was not as tight as in the previous cases, with the RFP value at full induction as high as 20% of the RFP value of the control. Taken together, in the range of interventions described above, the tuning of sgRNA affinity gave promising results with deletions and mismatches, which holds the potential to provide a wide range of diversity in the relative dissociation constants between dCas9:sgRNA and target DNA.

### Applications to Synthetic Circuit Design

**FIGURE 7:**
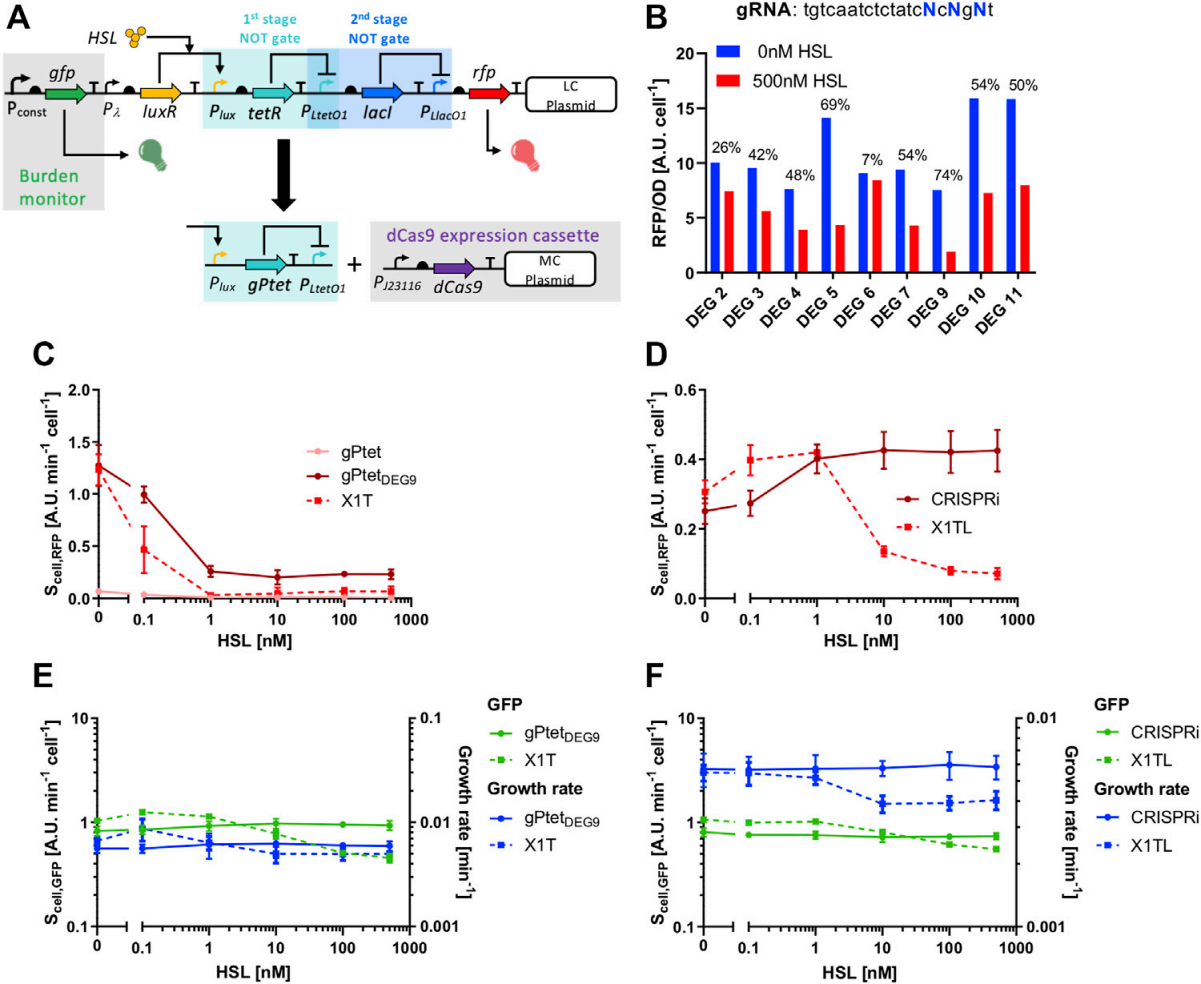
Fixing of a non-functional transcriptional cascade. **(A)** Architecture of the non-functional cascade, including the LuxR, TetR and LacI regulators; it has HSL as input and RFP as output, and the main source of failure was the high resource usage of the tetR module, highlighted in light blue and indicated as the first stage NOT gate. This module has been replaced with a CRISPRi logic inverter, including the parts illustrated below the black arrow. **(B)** Screening of nine degenerate gPtet guide variants without inducer or with 500 nM of HSL. The output is expressed as RFP/OD_600_. The numbers above the bars indicate the percent repression. **(C)** Characterization, in terms of RFP output, of the individual single-stage NOT gates with P_LtetO1_: X1T represents the NOT gate with TetR, gPtet indicates the CRISPRi NOT gate with the original gPtet guide, and DEG9 indicates the CRISPRi NOT gate with the selected gPtet variant, gPtet_DEG9_. **(D)** Characterization, in terms of RFP output, of the full transcriptional cascades: X1TL represents the original circuit for which an unexpected non-monotonically increasing HSL-dependent output was reported, and CRISPRi represents the fixed cascade with the gPtet_DEG9_ as repressor of the P_LtetO1_ promoter. **(E,F)** GFP and growth rate values for the same strains illustrated in panels **(C,D)**. In panels **(C,D)**, data are shown as the average RFP synthesis rate per cell; in panels **(E,F)**, data are shown as the average GFP synthesis rate per cell or average growth rate value. In panels **(C–F)**, data points represent the average value, with error bars representing the standard errors of the mean of at least three independent experiments.

Cell load could break the function of synthetic circuits, as it was previously reported (Qian et al., 2017; Pasotti et al., 2017). Here, we aim to exploit the low-burden properties of sgRNAs to fix a non-functional transcriptional cascade (Pasotti et al., 2017), in which the main responsible of cell load was *tetR* when expressed at high levels (Figures 7A–F). The considered cascade, assembled in low-copy vector, is named X1TL and includes an HSL-inducible device upstream of a TetR- and LacI-based NOT gates with RFP as circuit output (Figure 7A). Instead of showing a monotonically increasing HSL-dependent output, expected from the transfer functions of the individual inverter blocks, the circuit exhibited an increasing and then decreasing RFP output (Figure 7D). In the same work (Pasotti et al., 2017), a variant of this circuit was obtained which showed a functional output, increasing with HSL, by decreasing the strength of the *tetR* RBS. However, this approach is not always applicable, since RBS variations change the switch point of logic inverters, and a trade-off between the desired half-saturation constant and cell load may not exist. For this reason, we seek to use an sgRNA-based NOT gate to replace the TetR-based inverter with a new sgRNA-based NOT gate (Figure 7A). This approach was expected to fix the device without causing any input-dependent expression of resource-consuming components. A tuning of the gPtet repression strength was carried out using a single-stage NOT gate circuit in low-copy vector, in which an individual P_LtetO1_-targeting sgRNA was driven by an HSL-inducible module and expressed RFP as output under the control of P_LtetO1_. A similar circuit, called X1T and including *tetR* instead of the sgRNA, was used for comparison since it represented the desired transfer function of the NOT gate that had to be replaced by a CRISPRi module. As expected, the gPtet guide was characterized by a transfer function that was very different from the desired one of the TetR-based NOT gate, due to the previously observed low activity range (Figure 7C). An adjustment of the repression strength via truncated or mismatched gPtet variants was thereby necessary. Rational design of sgRNAs with desired features is currently a challenging genetic engineering task and screening steps are still necessary. From the data of the previous section, deletions seem to be promising because of the limited range of constructs that are needed to test their effect. Conversely, mismatches may require a higher number of plasmids to find a candidate with the desired properties, although computational tools have been proposed to support the prediction of mismatch effects (Farasat and Salis, 2016). Here, at first, five variants of gPtet were constructed by deleting nucleotides at their 5’ end. The resulting guides, with the truncation of 4, 7, 10, 11, 15 nucleotides, were tested but the resulting NOT gates did not result in a match with the desired transfer function due to a too low output range or too high basic activity (Supplementary Figure S10A). Interestingly, the expression of the 11-nt deleted gPtet was not tolerated by cells, which stopped growth upon the HSL addition at concentrations higher than 0.1 nM (data not shown). The toxicity of specific guides has already been reported, although this variant did not include any of the reported toxicity-related features (Cui et al., 2018). This effect has not been further investigated in this work and has been reported as a warning for future design interventions. For these reasons, despite the promising results obtained in the previous section for the P_luxRep_ model system, deletions failed to provide an sgRNA candidate with desired affinity. Then, a screening method was adopted, using degenerate primers, to obtain 20-nt gPtet variants with mismatches in three specific positions (Figure 7B). The screening included the per-cell measurement of RFP of several strains bearing the NOT gate with mismatched gPtet variants, among which the one with the high output range and low basic activity trade-off (named gPtet_DEG9_) was selected (Figure 7B) and sequenced. Its sequence was *tgt​caa​tct​cta​tcgcggat*, in which the degenerate nucleotides are underlined. When tested at different HSL concentrations, the individual logic inverter with gPtet_DEG9_ showed a transfer function that resembled the target one of the TetR-based inverter (Figure 7C), and also showed low cell load in terms of growth rate and GFP for any input value (Figure 7E). All the data reported for truncated and mismatched gPtet variants were relative to guides downstream of the wild-type P_lux_ promoter, thus including the three adenines in the transcribed region of the sgRNA. We also demonstrated that the removal of these three nucleotides did not result in relevant changes in the gPtet_DEG9_ transfer function (Supplementary Figure S10B). The final cascade including gPtet_DEG9_ was then constructed. It had an about 2-fold output range and it showed the expected monotonically increasing HSL-dependent output (Figure 7D). As expected, GFP was essentially independent from HSL, demonstrating that sgRNA expression caused no relevant burden (Figure 7F). On the other hand, the original circuit showed a GFP decrease for high HSL values, which corresponded to high TetR expression levels (Figure 7F). Despite the sgRNA-based cascade exhibited no HSL-dependent GFP or growth rate decrease, its GFP value obtained in the no-HSL condition was slightly lower than in the original cascade, suggesting that an additional load was present, most probably caused by multiple plasmids in the same strain, as previously observed (Pasotti et al., 2019), and not by dCas9 expression itself. Such effect was not observed in growth rate measurements (Figure 7F). It is worth noting that NOT gates having no expression-dependent resource usage are still an important achievement for synthetic circuit design to increase the chances of obtaining functional engineered strains.

**FIGURE 8:**
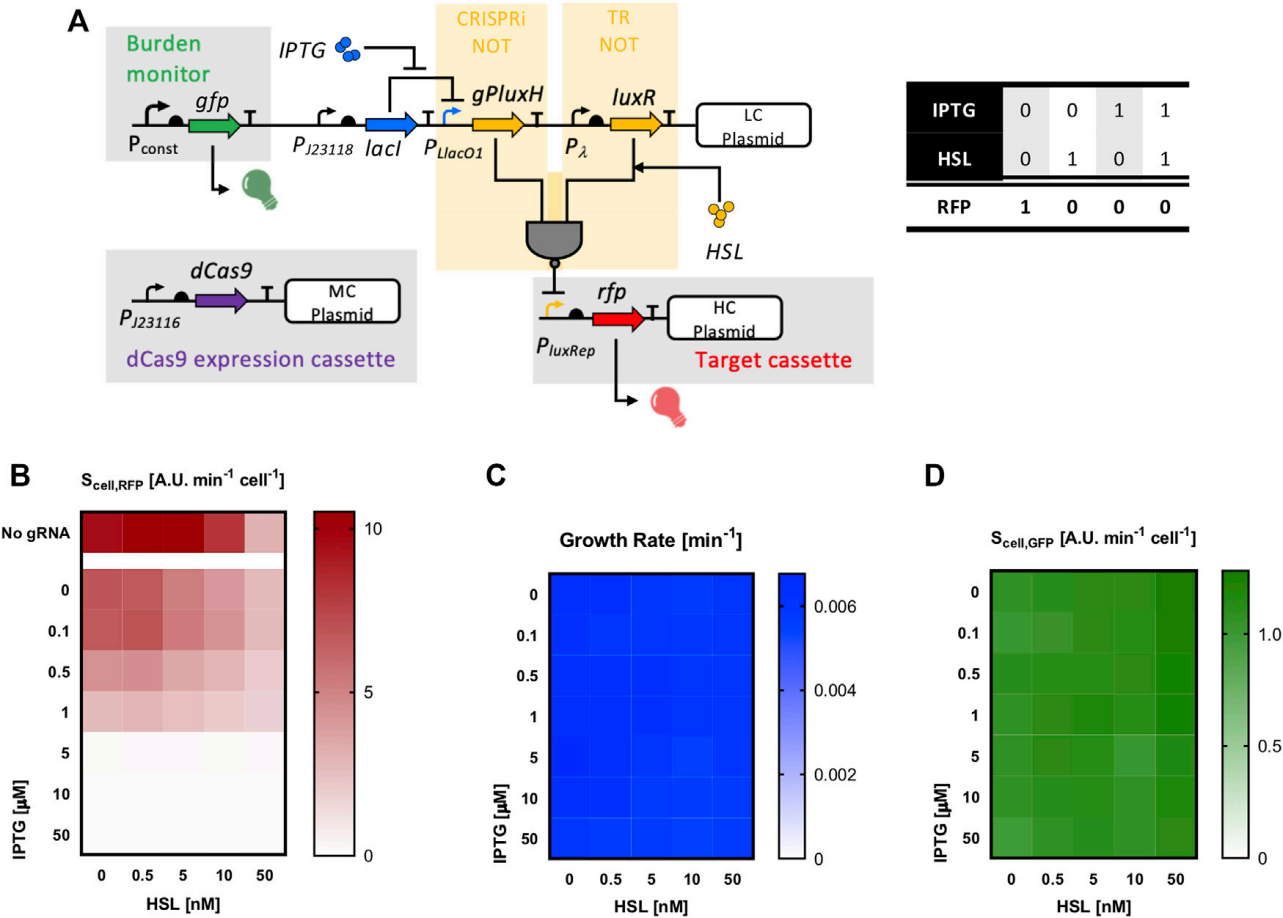
A NOR gate derived from a transcriptional regulator-based NOT gate and an additional CRISPRi logic inverter. **(A)** The HSL-dependent LuxR-based NOT gate (transcriptional regulator—TR—NOT) is shown in the upper-right and lower-right part of the panel, while the IPTG-dependent CRISPRi NOT gate is reported on the left. The resulting truth table with IPTG and HSL as inputs and with RFP as output is also reported. **(B–D)** RFP, growth rate and GFP values as a function of IPTG and HSL.

The use of programmable sgRNA-based NOT gates can be exploited not only to replace high resource usage repressors, but also to add regulatory modules to existing circuits, which is likely to cause no additional load for the cell except for possible plasmid-related load, as previously shown. We demonstrated a successful upgrade of an existing transcriptional regulator-based NOT gate by adding an sgRNA repressor, driven by another input, and converting the logic gate into a NOR gate (Figure 8A). The sgRNA and the LuxR repressor have highly overlapping DNA binding sites, likely to cause mutually exclusive binding events when both repressors are present. The engineering of a NOR gate is not *per se* a new achievement (Nielsen and Voigt, 2014), but its construction by upgrading an existing circuit, the exploitation of a transcriptional regulator and an sgRNA in the same logic gate, and the demonstration of obtaining a low-burden function are novel aspects that further contribute to showing the advantages of sgRNA logic inverters. The transcriptional regulator-based NOT gate had HSL as input and was composed by a constitutively expressed *luxR* gene in a low-copy vector and an RFP-expressing P_luxRep_ promoter in high-copy vector. An IPTG-inducible device, composed by a constitutive *lacI* expression cassette, was added in the low-copy vector to drive the sgRNA-based NOT gate, represented by the gPluxH guide. The dCas9 constitutive cassette was finally added as medium-copy plasmid, obtaining a three-plasmid strain. The RFP output in the resulting circuit could be repressed by either HSL or IPTG, or both. The RFP data, reported in Figure 8B and Supplementary Figure S11A, showed that the addition of the IPTG-dependent gPluxH cassette successfully implemented a 2-input NOR gate, with no relevant load as observed in the growth rate and GFP data (Figures 8C,D and Supplementary Figure S11B,C).

The RFP output of the two transcriptional cascades and of the NOR gate have also been studied *in silico*, using Hill equation models to compare theoretical and experimental behaviour of the constructed circuits. A description of the models is reported in Supplementary Text 1.2 for transcriptional cascades (1.2.1) and NOR gate (1.2.2). Fitting procedure from individual circuit components, data fitting results and estimated values are reported in Supplementary Text 1.2.3, Supplementary Figure S12 and Supplementary Table S2, respectively. Simulations, reported in Supplementary Figure S13, show that the models are able to capture the experimental data of the circuits.

## Conclusion

With the support of a large number of model systems, this study demonstrated the low-burden properties of sgRNA-based logic inverters for a wide range of repression values. Such modules were successfully applied to fix and upgrade two synthetic circuits. The former was a three-gene transcriptional cascade that was non-functional due to a high-resource consuming transcriptional regulator, which was replaced by a specific sgRNA that led to the desired function. The latter circuit was a transcriptional regulator-based NOT gate, which was turned into a NOR gate by programming a second input-controlled repression. In all the model systems investigated in this work, no relevant expression-dependent load was observed for sgRNAs. Nonetheless, sgRNA-based circuits may still be affected by load from target protein-coding genes, dCas9 expression and plasmid burden, all detected in some of the circuits investigated in this work. Ways to minimize such additional load are different: target proteins are application-specific and according to design specifications their expression may be decreased; plasmids are design-dependent and their presence could be minimized by additional DNA assembly and expression tuning without affecting the overall circuit function; a procedure to minimize the expression burden and toxicity of dCas9 has been herein reported, different from previous efforts, that could be adopted in future studies. Finally, the tuning of circuit transfer functions over a wide range of on/off values and switch points was demonstrated by several interventions, also supported by model simulations: changes of target DNA copy number, promoter engineering, sgRNA truncations, extensions and mismatches were tested as effective tuning methods in at least one case, although their success could depend on the specific circuit. Failures of such strategies, which have been observed in this work though not investigated in-depth, are worth mentioning: they included evolutionary instability of the circuit, toxicity of an expressed sgRNA, and requirement of random screening approaches to achieve the desired transfer function. Another issue that may be detrimental to circuit behaviour is the resource competition for dCas9, even though the conclusions of this work are expected to be valid for multiple sgRNAs, except for the low-burden dCas9 expression cassette that should be tuned, and specific methods to overcome such competition effect have been recently proposed. Mastering the design steps to overcome all the mentioned failure sources may further expand the potential of CRISPRi-based logic circuits.

## Data Availability

The raw data supporting the conclusion of this article will be made available by the authors, without undue reservation.

## Footnotes

1
http://partsregistry.org.

2
http://benchling.com.
