# Supplementary material for: CRISPR Interference Modules as Low-Burden Logic Inverters in Synthetic Circuits
Source: Front Bioeng Biotechnol. 2022 Jan 28;9:743950. doi: 10.3389/fbioe.2021.743950 (PMC8831695; doi:10.3389/fbioe.2021.743950)
Supplement: Supplementary file 1 [file DataSheet1.pdf]

frontiers

Supplementary Material

1 Supplementary Text

1.1 Background subtraction in fluorescence assays

Raw absorbance and red fluorescence time series acquired from microplate experiments were background-subtracted as previously reported (Pasotti et al., 2017) to obtain OD_{600} and RFP time series, proportional to the per-well cell density and fluorescent protein number. Sterile medium and a non-fluorescent TOP10 culture were used to measure absorbance and red fluorescence background, respectively. Since a significant cell density-dependent and growth rate-dependent autofluorescence was previously reported for GFP measurements in our experimental setup (Pasotti et al., 2017), green fluorescence was background-subtracted via a different procedure. We estimated the cell density- and growth rate-dependent autofluorescence as Eq.S1:

$$GFP_{auto}(t) = e^{q+m\cdot OD_{600}(t)} \quad (S1)$$

in which OD_{600} is the cell density of the culture, and *m* and *q* describe the linear relation between $\ln(OD_{600})$ and GFP autofluorescence level. The *m* and *q* coefficients are growth rate (μ)-dependent parameters, with a function defined as Eqs.S2-S3:

$$q(\mu) = \alpha_q + \beta_q \cdot \mu \quad (S2)$$
$$m(\mu) = \alpha_m + \beta_m \cdot \mu \quad (S3)$$

Therefore, the autofluorescence expression becomes (Eq.S4):

$$GFP_{auto}(t) \ = \ e^{\alpha_q+\beta_q\cdot\mu+(\alpha_m+\beta_m\cdot\mu)\cdot OD_{600}(t)} \quad (S4)$$

Using Eq.S4, the four coefficients were fitted from autofluorescence data of different cultures exhibiting diverse growth rates. The Matlab *regress* function was used for linear regression fitting in GFP_{auto} calculation. This expression served as a calibration curve providing autofluorescence values that are then subtracted from raw green fluorescence values, given OD_{600} and μ.

1.2 Mathematical modelling of transcriptional cascade and NOR gate

Hill equation models were defined for both circuits and parametrized by fitting experimental data from their individual components to eventually compare model prediction and final circuit behaviour.

1.2.1 Transcriptional cascade model description

A previously adopted steady-state model (Pasotti et al., 2017) was used to describe the HSL-dependent RFP output of the X1TL circuit (Eq.S5).

$$\begin{aligned}
S_{tet,max} &= \delta_{X1} + \frac{\alpha_{X1}}{1+\left(\frac{K_{X1}}{HSL}\right)^{\eta_{X1}}} \\
S_{lac,max} &= \delta_T + \frac{\alpha_T}{1+\left(\frac{T}{K_T}\right)^{\eta_T}} \\
S_{rfp,max} &= \delta_L + \frac{\alpha_L}{1+\left(\frac{L}{K_L}\right)^{\eta_L}} \\
D &= 1 + \Sigma_{X\lambda} + J_{tet} \cdot S_{tet,max} \\
T &= \frac{1}{(\gamma_{tet}+\mu)} \cdot \frac{S_{tet,max}}{D} \\
L &= \frac{1}{(\gamma_{lac}+\mu)} \cdot \frac{S_{lac,max}}{D} \\
S_{cell,X1TL} &= \frac{a}{(a+\mu)} \cdot \frac{S_{rfp,max}}{D}
\end{aligned} \qquad (S5)$$

Parameters have the same meaning and units as in previous work (Pasotti et al., 2017), also summarized in Supplementary Table S2. Briefly, T and L represent the intracellular concentrations of TetR and LacI; $S_{p,max}$ is the maximum synthesis rate of a generic regulated protein (p = TetR, LacI, RFP). Protein synthesis rate is modelled as a Hill function related to the activity of the upstream promoter (P_{lux}, P_{LtetO1} and P_{LlacO1}, respectively) with parameters δ, α, K and η, where $\delta + \alpha$ is the maximum expression rate, K is the input level corresponding to 50% of the expression rate range and η is the Hill coefficient; J_{tet} is the resource usage parameter of TetR; the cell load caused by LacI and RFP is assumed to be negligible at the expression levels spanned in the cascade circuit (Pasotti et al., 2017); $\Sigma_{X\lambda}$ is the additional load given by the constitutive expression of LuxR. The term D is the scale factor between the maximum synthesis rate of a protein into the actual synthesis rate, obtained as the sum of cell load-related factors. The a, μ, γ_{tet} and γ_{lac} represent the RFP maturation rate, cell growth rate, TetR and LacI protein degradation rates, respectively.

A model predicting the HSL-dependent output of the CRISPRi transcriptional cascade was derived from Eq.S5 by considering the $gPtet_{DEG9}$:dCas9 repressor (named *Cdeg9*) instead of TetR, under the following assumptions: the cell load caused by the expression of sgRNA is negligible; the intracellular $gPtet_{DEG9}$:dCas9 repressor complex was modeled, without describing the constitutive expression of dCas9 and its further binding to $gPtet_{DEG9}$; the dilution rate of the CRISPRi repressor complex is equal to cell growth rate (Qi et al., 2013); an additional parameter (Σ_C) is used in D to account for the GFP value that is slightly lower in the CRISPRi cascade compared with X1TL for HSL = 0 (Figure 7F), probably due to the presence of an additional plasmid, maintained at medium copy, that may represent a load for the cells (Pasotti et al., 2019). The resulting equations are reported in the Eq.S6 system.

$$S_{deg9,max} = \delta_{X1} + \frac{\alpha_{X1}}{1 + \left(\frac{K_{X1}}{HSL}\right)^{\eta_{X1}}}$$
$$S_{lac,max} = \delta_{deg9} + \frac{\alpha_{deg9}}{1 + \left(\frac{Cdeg9}{K_{deg9}}\right)^{\eta_{deg9}}}$$
$$S_{rfp,max} = \delta_L + \frac{\alpha_L}{1 + \left(\frac{L}{K_L}\right)^{\eta_L}} \qquad (S6)$$
$$D = 1 + \Sigma_{X\lambda} + \Sigma_C$$
$$Cdeg9 = \frac{S_{deg9,max}}{D \cdot \mu}$$
$$L = \frac{S_{lac,max}}{D \cdot (\gamma_{lac} + \mu)}$$
$$S_{cell,CRISPRi} = \frac{a}{(a + \mu)} \cdot \frac{S_{rfp,max}}{D}$$

1.2.2 NOR gate model description

A kinetic model of the main regulatory steps occurring in the NOR gate circuit was defined (Eq.S7).

$$C_{rep} + D_F \overset{K_C}{\leftrightarrow} D_{R1}$$
$$HSL + R_2 \overset{K_H}{\leftrightarrow} Q$$
$$Q + D_F \overset{K_R}{\leftrightarrow} D_{R2}$$
$$C_{tot} = \frac{1}{\mu} \cdot \left(\delta_I + \frac{\alpha_I}{1 + \left(\frac{K_I}{IPTG}\right)^{\eta_I}} \right)$$
$$D_{tot} = D_F + D_{R1} + D_{R2}$$
$$C_{tot} = C_{rep} + D_{R1} \qquad (S7)$$
$$R_{2T} = R_2 + Q + D_{R2}$$
$$D_F = \frac{D_{tot}}{1 + \frac{C_{tot}}{K_C} + \frac{R_{2T}/K_R}{1 + \frac{K_H}{HSL}}}$$
$$S_{cell,NOR} = \frac{a}{(a + \mu)} \cdot \sigma \cdot D_F = \frac{a}{(a + \mu)} \cdot \frac{\beta}{1 + \frac{C_{tot}}{K_C} + \frac{U}{1 + \frac{K_H}{HSL}}}$$

The model describes the binding of the gPluxH:dCas9 repressor complex (C_{rep}) to the unoccupied P_{luxRep} promoter sequence (D_F) obtaining the promoter in a repressed state (D_{R1}), the binding of HSL to a LuxR dimer (R_2) obtaining the HSL-LuxR complex (Q), the binding of Q to D_F obtaining the promoter in a second repressed state (D_{R2}), and the RFP synthesis rate per cell ($S_{cell,NOR}$) as proportional to D_F (with proportionality constant $\sigma \cdot a/(a+\mu)$). The symbols on the bidirectional arrows of the reactions indicate the resulting dissociation equilibrium constants. Conservation laws are also defined

in Eq.S7 for total DNA, repressor and LuxR in cells. A steady-state solution for the free promoter D_F was derived under the following assumptions: no significant cell load affects the recombinant strain; the total intracellular amount of repressors (C_{tot} and R_{2T}) is much higher than the target DNA concentration; the binding events of C_{rep} and Q to the free promoter are mutually exclusive due to the short distance between the CRISPRi target site and the lux box; the activity of the P_{luxRep} promoter in the repressed state is null; C_{rep} is approximated by an IPTG-dependent Hill equation. In the final expression of $S_{cell,NOR}$, the lumped parameters $\beta = \sigma \cdot D_F$ and $U = R_{2T}/K_R$ are present. A summary of parameter values and units is reported in Supplementary Table S2.

1.2.3 Model implementation and fitting procedure

The models were implemented via Matlab R2017b (Mathworks, Natick, MA). Implicit equations (Eq.S5) were solved with a custom script implementing the fixed point method as it was carried out previously (Pasotti et al., 2017). The *lsqnonlin* routine, implementing the least squares algorithm, was used to fit experimental data (average values) to estimate the unknown parameters. For the transcriptional cascade, δ_{deg9}, α_{deg9}, K_{deg9} and η_{deg9} were estimated by fitting the RFP data of the $gPtet_{DEG9}$ NOT gate (Figure 7B-C) using the model in Eq.S8.

$$\begin{gathered}
S_{deg9,max} = \delta_{X1} + \frac{\alpha_{X1}}{1 + \left(\frac{K_{X1}}{HSL}\right)^{\eta_{X1}}} \\
S_{rfp,max} = \delta_{deg9} + \frac{\alpha_{deg9}}{1 + \left(\frac{Cdeg9}{K_{deg9}}\right)^{\eta_{deg9}}} \\
D = 1 + \Sigma_{X\lambda} + \Sigma_C \\
Cdeg9 = \frac{S_{deg9,max}}{D \cdot \mu} \\
S_{cell} = \frac{a}{(a + \mu)} \cdot \frac{S_{rfp,max}}{D}
\end{gathered} \qquad (S8)$$

The Σ_C parameter was computed by solving $\frac{1+\Sigma_{X\lambda}}{1+\Sigma_{X\lambda}+\Sigma_C} = \frac{S_{cell,GFP}^{CRISPRi}}{S_{cell,GFP}^{X1TL}}$ using the GFP data in Figure 7F for HSL = 0.

For the NOR gate, the β parameter was computed as $((a+\mu)/a) \cdot RFP_{control}$, where $RFP_{control}$ is the RFP output value of the control strain in Figure 5H for HSL=0; the RFP data of the No gPluxH condition (Figure 8B) were fitted with the $S_{cell,NOR}$ equation for $C_{tot} = 0$ to estimate the U and K_H parameters; the RFP data of the IPTG/P_{LlacO1}-inducible system (Supplementary Figure S5) and of the IgLUX circuit (Figure 5H) were simultaneously fitted with Eq.S9 to estimate the δ_I, α_I, K_I, η_I and K_C parameters.

$$S_{cell,PLlacO1} = \frac{a}{(a+\mu)} \cdot \left(\delta_I + \frac{\alpha_I}{1 + \left(\frac{K_I}{IPTG}\right)^{\eta_I}} \right)$$

$$C_{tot} = \frac{1}{\mu} \cdot \left(\delta_I + \frac{\alpha_I}{1 + \left(\frac{K_I}{IPTG}\right)^{\eta_I}} \right) \qquad (S9)$$

$$S_{cell,IgLUX} = \frac{a}{(a+\mu)} \cdot \frac{\beta}{1 + \frac{C_{tot}}{K_C}}$$

A growth rate value was fixed for both fitting and simulations as the typical value measured in the two strains bearing the cascade and the NOR gate.

2 Supplementary Figures and Tables

2.1 Supplementary Figures

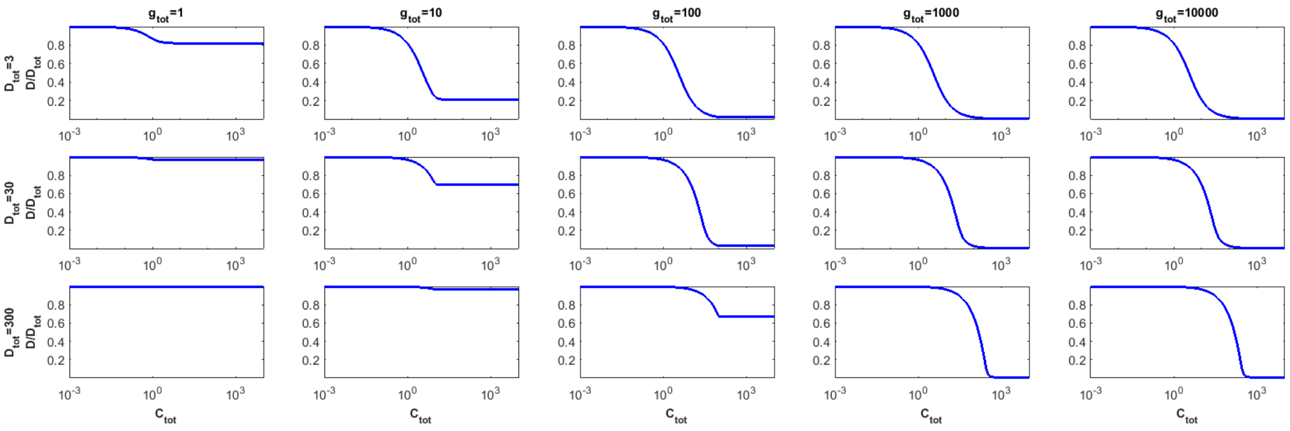

Supplementary Figure S1. Mathematical model simulations of circuit output as a function of dCas9, sgRNA and target DNA copy number. The output curves represent the intracellular concentration of free promoter DNA (*D*) normalized by the total concentration of available promoter DNA (D_{tot}). The independent variable of the simulations is the intracellular concentration of dCas9 (C_{tot}, expcessed as nM concentration). Simulations are shown for different values of sgRNA (g_{tot}, expressed as nM concentration, in the columns) and DNA (D_{tot}, expressed as nM concentration, in the rows). Parameters: K_1 = 0.3 nM, K_2 = 2 nM.

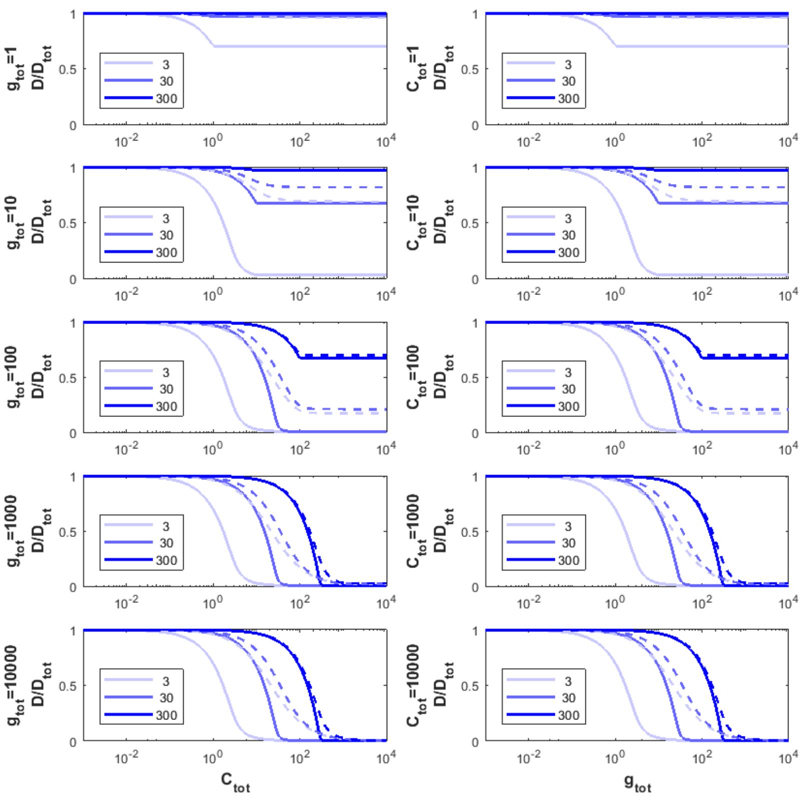

Supplementary Figure S2. Mathematical model simulations of circuit output as a function of dCas9, sgRNA and target DNA copy number. The output curves represent the intracellular concentration of non-repressed promoter DNA (D) normalized by the total concentration of available promoter DNA (D_{tot}). The independent variable of the simulations is either the intracellular concentration of dCas9 (C_{tot}, expcessed as nM concentration, panels in the left column) or the intracellular concentration of sgRNA (g_{tot}, expcessed as nM concentration, panels in the right column). Simulations are shown for different values of sgRNA (left) or dCas9 (right), and DNA (D_{tot}, expressed as nM concentration) as different curves in each panel. Every panel reports six curves, corresponding to three different D_{tot} values, as indicated in the legend, and two different parameter sets (solid line: K_1 = 0.03 nM, K_2 = 0.2 nM; dashed line: K_1 = 3 nM, K_2 = 20 nM).

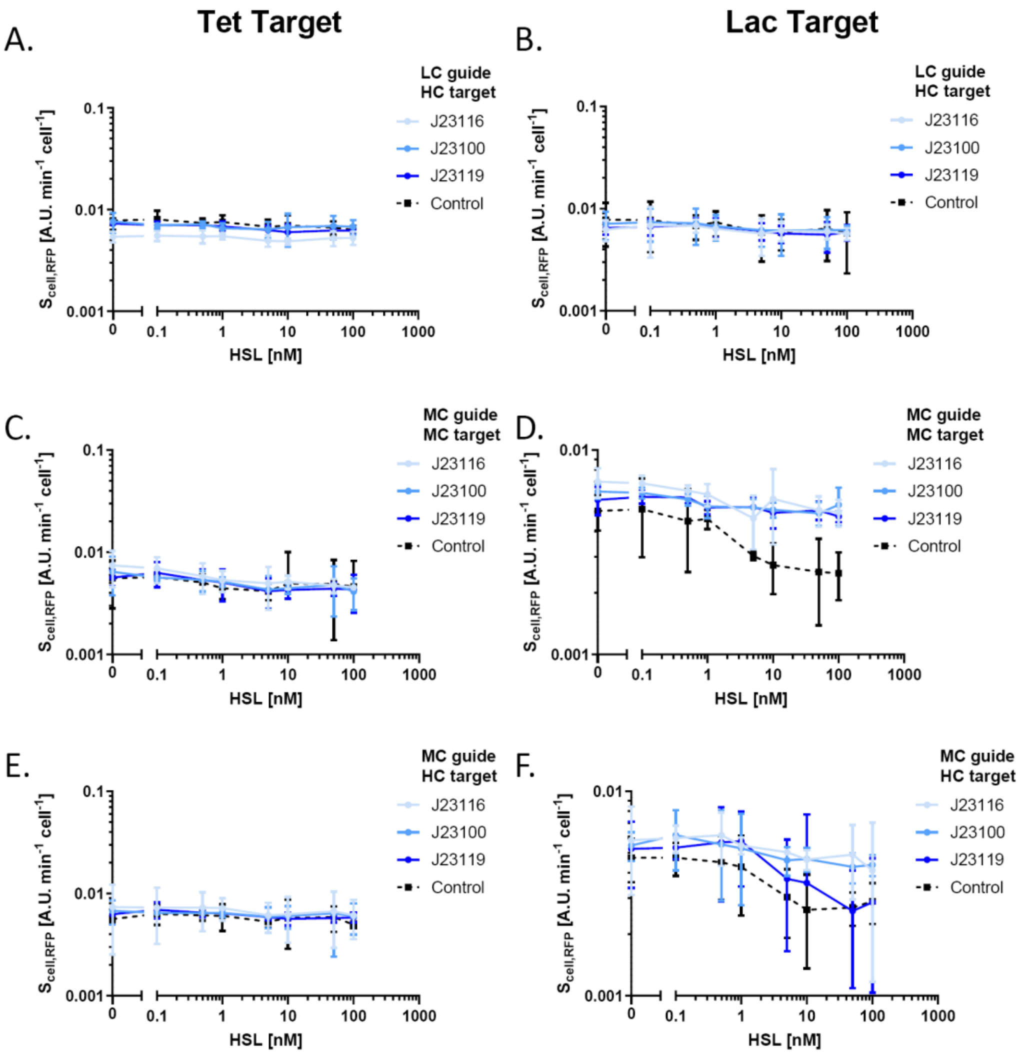

Supplementary Figure S3. Growth rate values of recombinant strains with HSL-inducible dCas9 and constitutive sgRNA. Data are reported as a function of HSL. In each panel, the copy number of the sgRNA constitutive cassette (low copy – LC, medium copy – MC) and the copy number of the target (medium copy – MC, high copy – HC) are reported. Two different targeting systems (Tet – panels A, C, E, and Lac – panels B, D, F) are reported: gPtet and gPlac, which repress the P_{LtetO1} and P_{LlacO1} promoters, respectively, that drive RFP. Each panel includes four curves: three of them correspond to circuits with the sgRNA under the control of three different constitutive promoters of diverse strengths (weak, medium and strong for J23116, J23100 and J23119, respectively), and one curve corresponds to a non-specific targeting control in which the medium-strength J23100 promoter constitutively transcribes a non-targeting sgRNA: gPlac and gPtet for the P_{LtetO1} and P_{LlacO1} promoters in the Tet and Lac systems, respectively. Data points represent the average value and error bars represent the standard errors of the mean of at least 3 independent experiments.

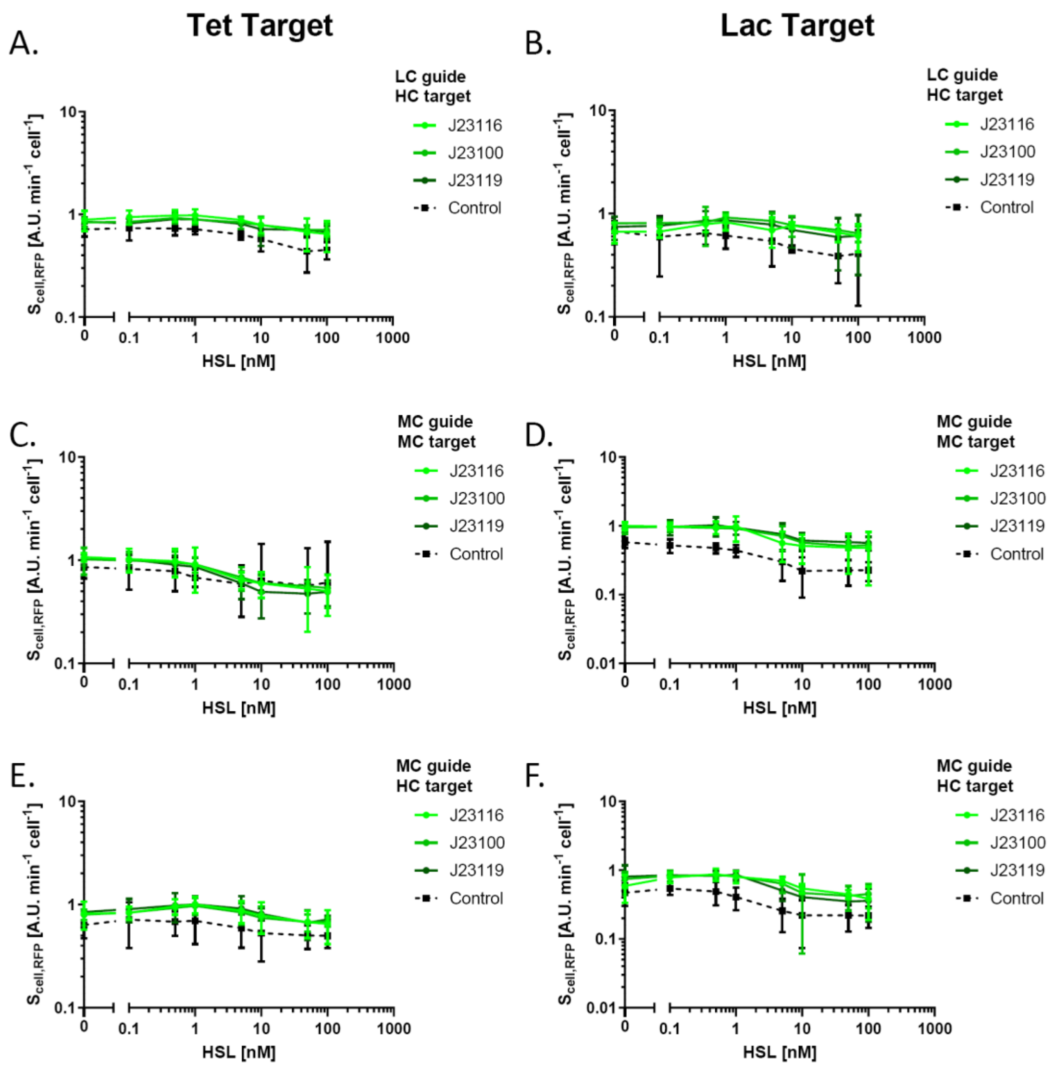

Supplementary Figure S4. GFP values of recombinant strains with HSL-inducible dCas9 and constitutive sgRNA. Data are reported as a function of HSL. In each panel, the copy number of the sgRNA constitutive cassette (low copy – LC, medium copy – MC) and the copy number of the target (medium copy – MC, high copy – HC) are reported. Two different targeting systems (Tet – panels A, C, E, and Lac – panels B, D, F) are reported: gPtet and gPlac, which repress the P_{LtetO1} and P_{LlacO1} promoters, respectively, that drive RFP. Each panel includes four curves: three of them correspond to circuits with the sgRNA under the control of three different constitutive promoters of diverse strengths (weak, medium and strong for J23116, J23100 and J23119, respectively), and one curve corresponds to a non-specific targeting control in which the medium-strength J23100 promoter constitutively transcribes a non-targeting sgRNA: gPlac and gPtet for the P_{LtetO1} and P_{LlacO1} promoters in the Tet and Lac systems, respectively. Data points represent the average value and error bars represent the standard errors of the mean of at least 3 independent experiments.

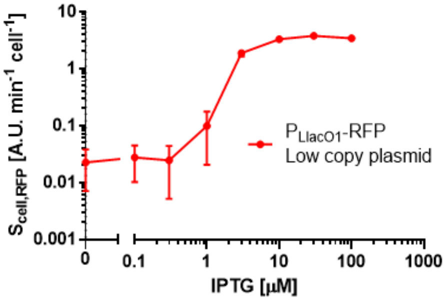

Supplementary Figure S5. Transfer function, with RFP as output, of the IPTG-inducible system including P_{LlacO1}. The Ir recombinant strain was used. Data are shown as the average RFP synthesis rate per cell, as a function of IPTG. Data points represent the average value and error bars represent the standard errors of the mean of at least 3 independent experiments.

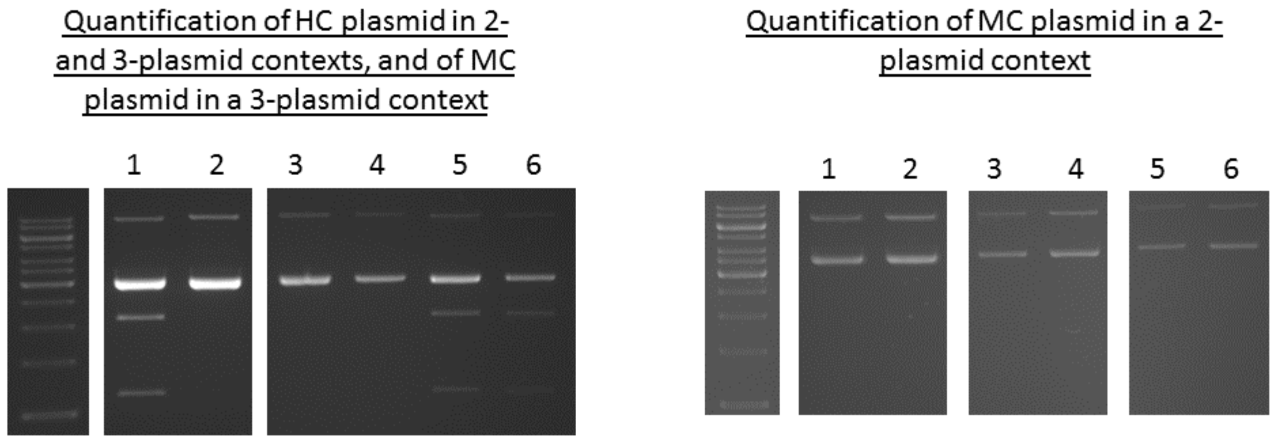

Supplementary Figure S6. Copy number quantification in recombinant strains with two or more plasmids. All of them include a low-copy vector (highest molecular weight band). The medium- and high-copy vector amounts were quantified relative to the intensity of the low-copy vector. Electrophoresis gel (1% agarose with TBE) pictures, with ethidium bromide staining, are shown for strains $H_{-3}dgLAC_{100,LC}P_{tet,HC}$ and $H_{-3}dgLAC_{100,MC}P_{tet,HC}$ (gel on the left) and $H_{-3}dgLAC_{100,MC}P_{tet,MC}$ (gel on the right). Gel on the left: lanes 1, 5, 6 contain the $H_{-3}dgLAC_{100,MC}P_{tet,HC}$ plasmids at different dilutions, digested with *XbaI-SacII* (*XbaI* is a single cutter in all the three plasmids and *SacII* is a single cutter only in the MC plasmid); lanes 2, 3, 4 contain the $H_{-3}dgLAC_{100,LC}P_{tet,HC}$ plasmids at different dilutions, digested with *XbaI-SacII* (*XbaI* is a single cutter in all the three plasmids and *SacII* does not cut any of the plasmids). Gel on the right: lanes 1, 3, 5 contain the $H_{-3}dgLAC_{100,MC}P_{tet,MC}$ plasmids at different dilutions, digested with *XbaI* (single cutter in both plasmids); lanes 2, 4, 6 are analogous to lanes 1, 3, 5, but they are relative to a biological replicate. The DNA ladder is GeneRuler 1 Kb (Thermo Scientific), with the lowest band reported in the pictures corresponding to the 1000-bp size.

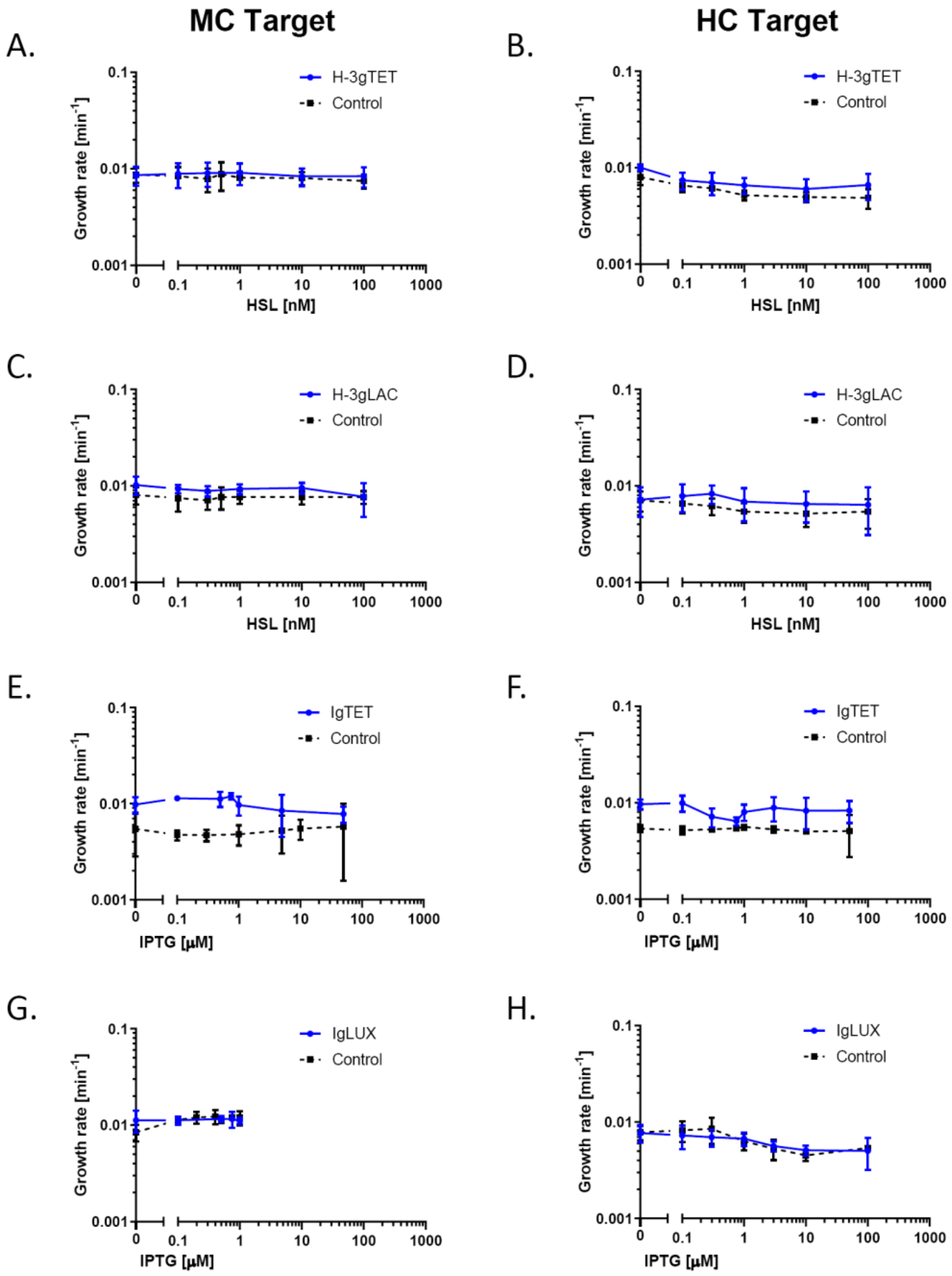

Supplementary Figure S7. Growth rate values of recombinant strains with constitutive dCas9 and inducible sgRNA. Data are reported as a function of the inducer concentration driving sgRNA expression (HSL, in panels A-D, or IPTG, in panels E-H). In each panel, the CRISPRi targeting system is reported as gPtet, gPlac and gPluxH, which repress the P_{LtetO1}, P_{LlacO1} and P_{luxRep} target promoters, respectively, that drive RFP. Two different copy number contexts for the target are reported: medium copy (MC) and high copy (HC). Each panel includes two curves, corresponding to circuits with specific or non-specific targeting system. The latter is referred to as control and the used sgRNAs are gPlac (panels A-B), gPtet (panels C-D and G-H) and gPluxH (E-F). Data points represent the average value and error bars represent the standard errors of the mean of at least 3 independent experiments.

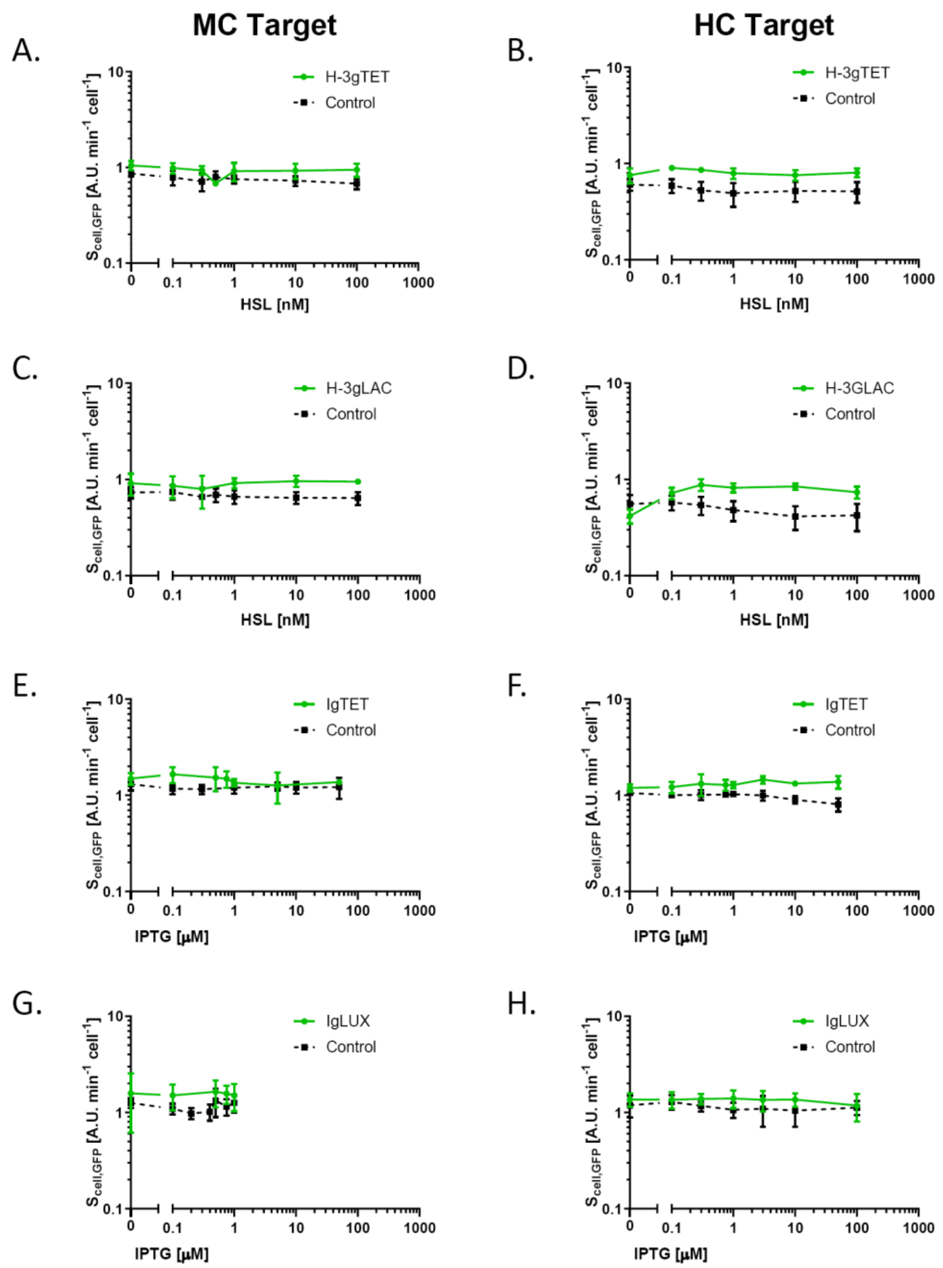

Supplementary Figure S8. GFP values of recombinant strains with constitutive dCas9 and inducible sgRNA. Data are reported as a function of the inducer concentration driving sgRNA expression (HSL, in panels A-D, or IPTG, in panels E-H). In each panel, the CRISPRi targeting system is reported as gPtet, gPlac and gPluxH, which repress the P_{LtetO1}, P_{LlacO1} and P_{luxRep} target promoters, respectively, that drive RFP. Two different copy number contexts for the target are reported: medium copy (MC) and high copy (HC). Each panel includes two curves, corresponding to circuits with specific or non-specific targeting system. The latter is referred to as control and the used sgRNAs are gPlac (panels A-B), gPtet (panels C-D and G-H) and gPluxH (E-F). Data points represent the average value and error bars represent the standard errors of the mean of at least 3 independent experiments.

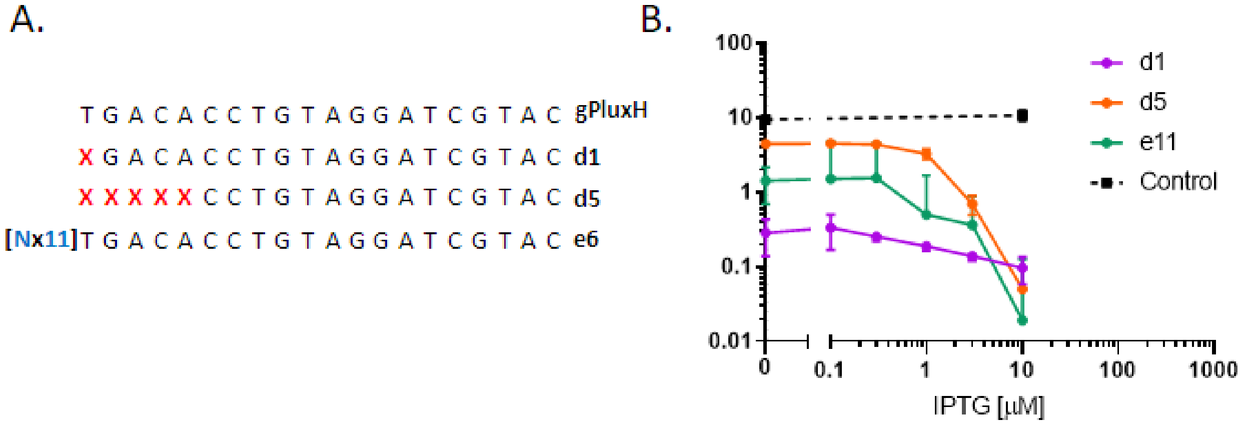

Supplementary Figure S9. gPluxH variants not reported in the main text. (A) Description of the gPluxH variants. Blue nucleotides represent mismatches compared with the target sequence. (B) Transfer functions, with RFP as output, are reported as a function of IPTG concentration, driving the gPluxH variants expression in different circuits with constitutive dCas9, IPTG-inducible sgRNA, and P_{luxRep} as target driving RFP in a medium copy plasmid. The control represents an identical circuit but including the gPtet guide, which is not able to target P_{luxRep}. Data are shown as the average RFP synthesis rate per cell and data points represent the average value, with error bars representing the standard errors of the mean of at least 3 independent experiments.

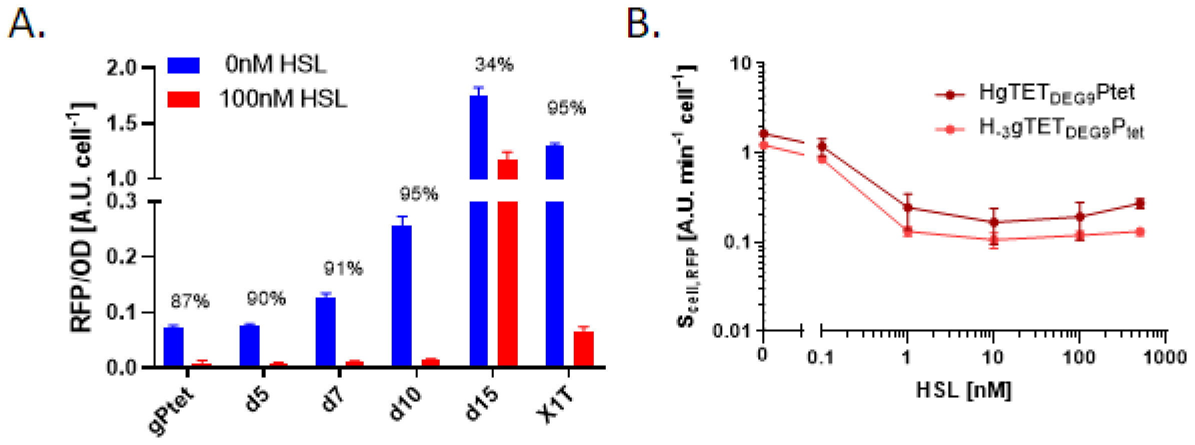

Supplementary Figure S10. gPtet variants not reported in the main text. (A) sgRNAs with truncations and their effect on the HSL-inducible sgRNA circuit with constitutive dCas9 in MC and target in LC (HgTET$_{d[n]}$P$_{tet}$ strains). All of them are transcribed with the three adenines present in the wild-type P$_{lux}$ promoter. The number of deleted nucleotides at the 5' end of the guide is reported and the bars show the RFP output in the no induction and full induction conditions. The numbers above the bars indicate the percent repression. (B) Comparison between the gPtetDEG9 variant when expressed by the wild-type P$_{lux}$ and the modified P$_{lux\text{-}3A}$ promoters (HgTET$_{DEG9}$P$_{tet}$ and H$_{-3}$gTET$_{DEG9}$P$_{tet}$ strains, respectively). Data are shown as the average RFP synthesis rate per cell and data points and bars represent the average value, with error bars representing the standard errors of the mean of at least 3 independent experiments.

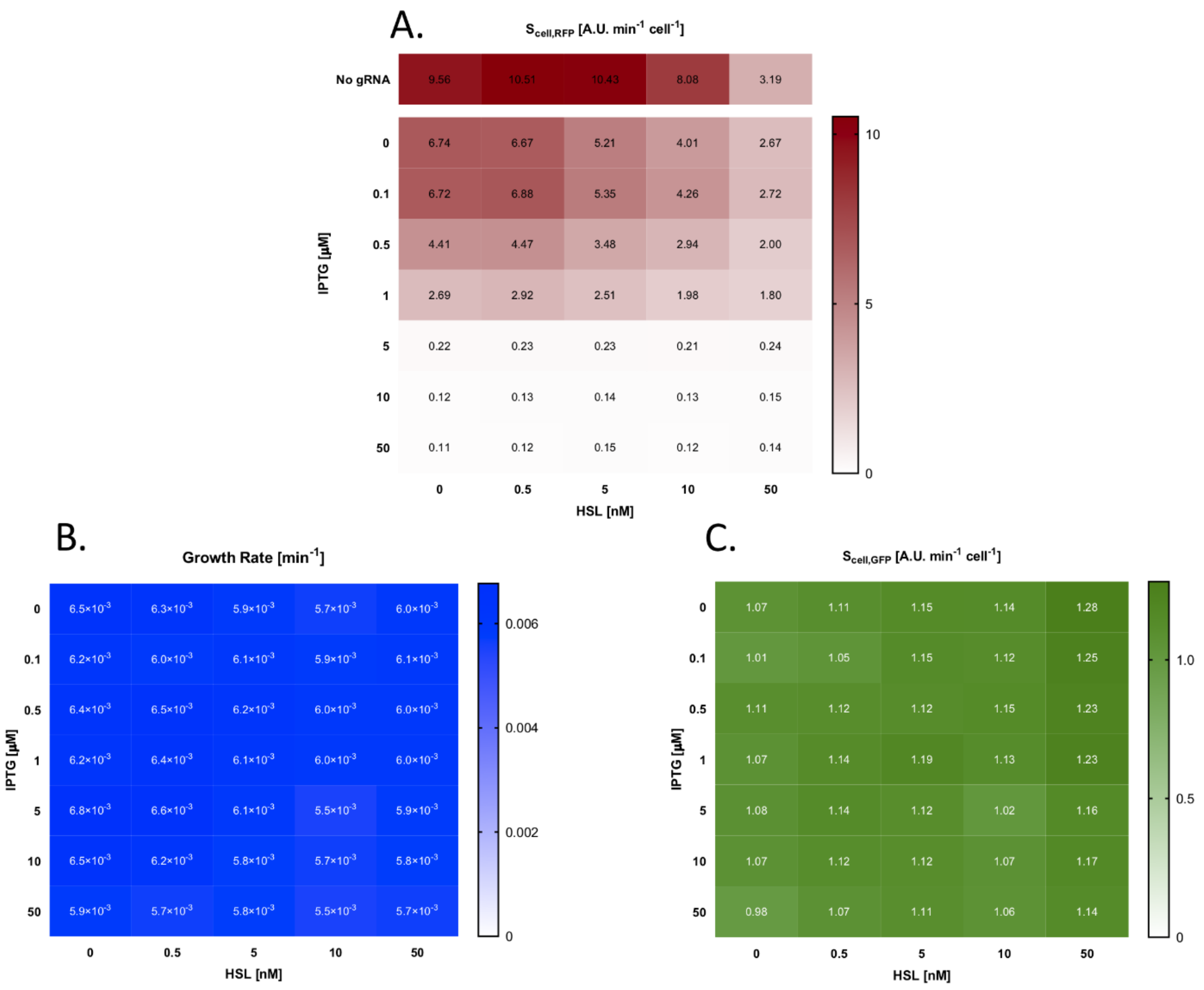

Supplementary Figure S11. NOR gate characterization. (A-C) Heatmaps and individual mean values of RFP, growth rate and GFP as a function of IPTG and HSL.

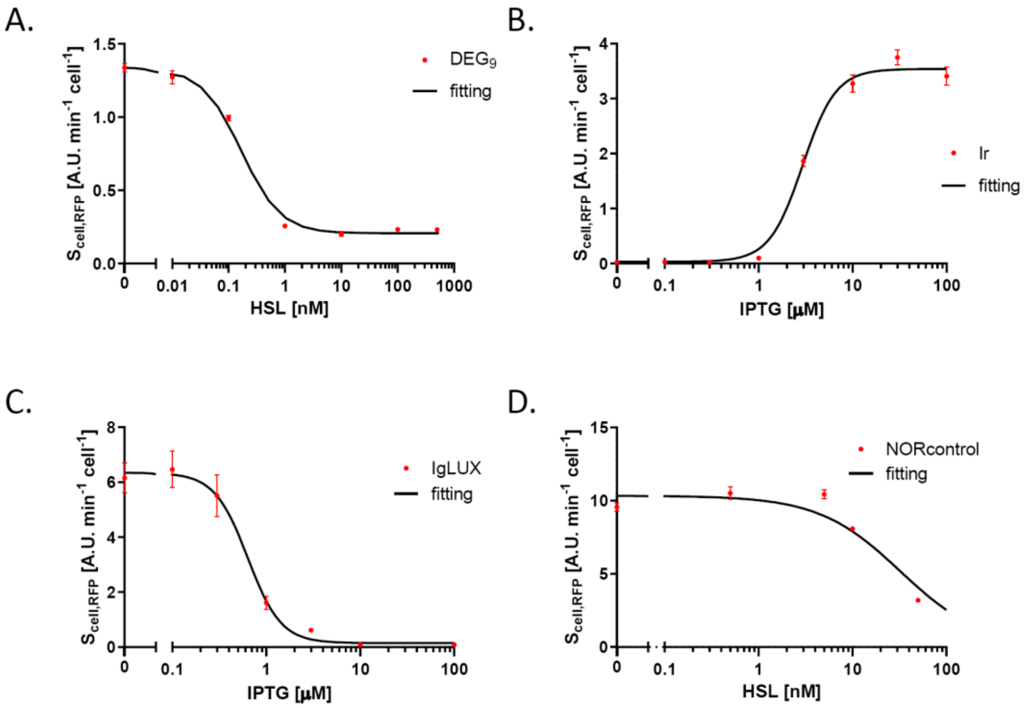

Supplementary Figure S12. Fitting of RFP data for the estimation of model parameters. Fitting of the (A) HSL-inducible $gPtet_{DEG9}$ NOT gate data ($HgTET_{DEG9}P_{tet}$ strain), (B) IPTG-inducible P_{LlacO1} data (Ir strain), (C) IPTG-inducible gPluxH NOT gate data with target promoter in high-copy plasmid ($IgLUXd_{116}P_{luxRep,HC}$ strain), (D) HSL-repressible P_{luxRep} promoter (NORcontrol strain). Data points represent the average data used for the fitting procedure and solid lines represent the fitted curves.

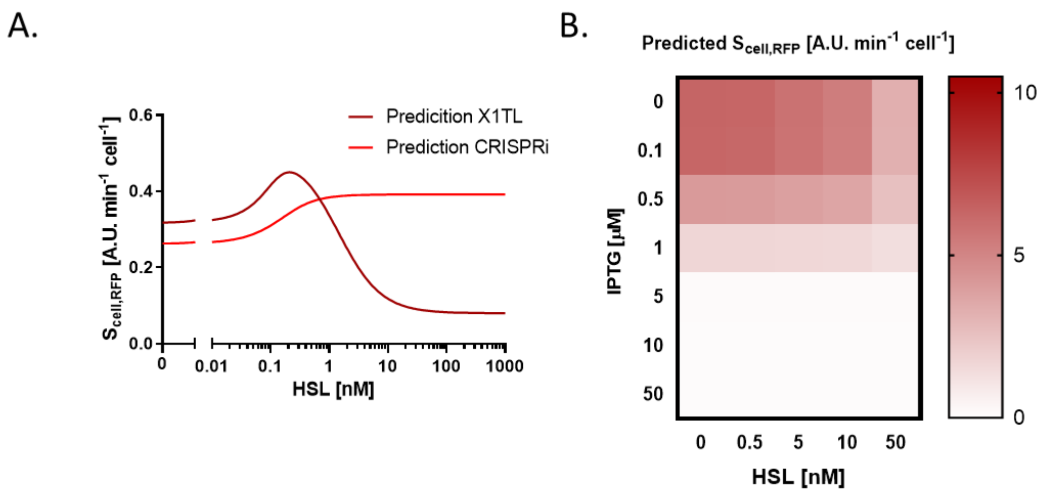

Supplementary Figure S13. Model simulations to predict the RFP output of the transcriptional cascades and the NOR gate. (A) Cascades. (B) NOR gate. The two graphs are consistent with the ones in Figure 7D and 8B, in which experimental data are reported.

2.2 Supplementary Tables

Supplementary Table S1. Description of the recombinant strains used in this work. All the plasmids are reported according with the nomenclature of the Registry of Standard Biological Parts, with the "BBa_" prefix omitted. Unless differently indicated, all the parts in the low-, medium- and high-copy plasmids are in the pSB4C5, pSB3K3 and pSB1A2 vector backbones.

Strain	Description	Low-copy plasmid	Medium-copy plasmid	High-copy plasmid
Hr	HSL-inducible RFP in low-copy plasmid under the control of P_{lux}	J107223	-	-
$H_{-3}r$	HSL-inducible RFP in low-copy plasmid under the control of $P_{lux\text{-}3A}$	J107224	-	-
Hd	HSL-inducible dCas9 in low-copy plasmid under the control of P_{lux}	J107225	-	-
$H_{-3}d$	HSL-inducible dCas9 in low-copy plasmid under the control of $P_{lux\text{-}3A}$	J107216	-	-
116d	Constitutive dCas9 cassette in medium-copy plasmid	-	J107202	-
Ir	IPTG-inducible RFP in low-copy plasmid under the control of P_{LlacO1}	J107211	-	-
J101R	Reference strain for RFP fluorescence	J107029	-	-
J101G	Reference straini for GFP fluorescence	K173001	-	-
Constructs with inducible dCas9 and constitutive sgRNA				
$H_{-3}dgTET_{116,LC}P_{tet,HC}$	HSL-inducible dCas9 in low-copy plasmid with weak constitutive gPtet in low-copy and the target P_{LtetO1} promoter driving RFP in high-copy	J107217	-	I13521
$H_{-3}dgTET_{100,LC}P_{tet,HC}$	HSL-inducible dCas9 in low-copy plasmid with medium-strength constitutive gPtet in low-copy and the target P_{LtetO1} promoter driving RFP in high-copy	J107218	-	I13521
$H_{-3}dgTET_{119,LC}P_{tet,HC}$	HSL-inducible dCas9 in low-copy plasmid with strong constitutive gPtet in low-copy and the target P_{LtetO1} promoter driving RFP in high-copy	J107219	-	I13521
$H_{-3}dgTET_{116,MC}P_{tet,MC}$	HSL-inducible dCas9 in low-copy plasmid with weak constitutive gPtet in medium-copy and the target P_{LtetO1} promoter driving RFP in medium-copy	J107216	J107237	-
$H_{-3}dgTET_{100,MC}P_{tet,MC}$	HSL-inducible dCas9 in low-copy plasmid with medium-strength constitutive gPtet in medium-copy and the target P_{LtetO1} promoter driving RFP in medium-copy	J107216	J107238	-
$H_{-3}dgTET_{119,MC}P_{tet,MC}$	HSL-inducible dCas9 in low-copy plasmid with strong constitutive gPtet in medium-copy and the target P_{LtetO1} promoter driving RFP in medium-copy	J107216	J107239	-

$H_{-3}dgTET_{116,MC}P_{tet,HC}$	HSL-inducible dCas9 in low-copy plasmid with weak constitutive gPtet in medium-copy and the target P_{LtetO1} promoter driving RFP in high-copy	J107216	J107213	I13521
$H_{-3}dgTET_{100,MC}P_{tet,HC}$	HSL-inducible dCas9 in low-copy plasmid with medium-strength constitutive gPtet in medium-copy and the target P_{LtetO1} promoter driving RFP in high-copy	J107216	J107214	I13521
$H_{-3}dgTET_{119,MC}P_{tet,HC}$	HSL-inducible dCas9 in low-copy plasmid with strong constitutive gPtet in medium-copy and the target P_{LtetO1} promoter driving RFP in high-copy	J107216	J107215	I13521
$H_{-3}dgLAC_{100,LC}P_{tet,HC}$	Non-specific targeting control for the $H_{-3}dgTET_{1XX,LC}P_{tet,HC}$ strains	J107221	-	I13521
$H_{-3}dgLAC_{100,MC}P_{tet,MC}$	Non-specific targeting control for the $H_{-3}dgTET_{1XX,MC}P_{tet,MC}$ strains	J107216	J107240	-
$H_{-3}dgLAC_{100,MC}P_{tet,HC}$	Non-specific targeting control for the $H_{-3}dgTET_{1XX,MC}P_{tet,HC}$ strains	J107216	J107208	I13521
$H_{-3}dgLAC_{116,LC}P_{lac,HC}$	HSL-inducible dCas9 in low-copy plasmid with weak constitutive gPlac in low-copy and the target P_{LlacO1} promoter driving RFP in high-copy	J107220	-	J107010
$H_{-3}dgLAC_{100,LC}P_{lac,HC}$	HSL-inducible dCas9 in low-copy plasmid with medium-strength constitutive gPlac in low-copy and the target P_{LlacO1} promoter driving RFP in high-copy	J107221	-	J107010
$H_{-3}dgLAC_{119,LC}P_{lac,HC}$	HSL-inducible dCas9 in low-copy plasmid with strong constitutive gPlac in low-copy and the target P_{LlacO1} promoter driving RFP in high-copy	J107222	-	J107010
$H_{-3}dgLAC_{116,MC}P_{lac,MC}$	HSL-inducible dCas9 in low-copy plasmid with weak constitutive gPlac in medium-copy and the target P_{LlacO1} promoter driving RFP in medium-copy	J107216	J107231	-
$H_{-3}dgLAC_{100,MC}P_{lac,MC}$	HSL-inducible dCas9 in low-copy plasmid with medium-strength constitutive gPlac in medium-copy and the target P_{LlacO1} promoter driving RFP in medium-copy	J107216	J107232	-
$H_{-3}dgLAC_{119,MC}P_{lac,MC}$	HSL-inducible dCas9 in low-copy plasmid with strong constitutive gPlac in medium-copy and the target P_{LlacO1} promoter driving RFP in medium-copy	J107216	J107233	-
$H_{-3}dgLAC_{116,MC}P_{lac,HC}$	HSL-inducible dCas9 in low-copy plasmid with weak constitutive gPlac in medium-copy and the target P_{LlacO1} promoter driving RFP in high-copy	J107216	J107207	J107010
$H_{-3}dgLAC_{100,MC}P_{lac,HC}$	HSL-inducible dCas9 in low-copy plasmid with medium-strength constitutive gPlac in medium-copy and the target P_{LlacO1} promoter driving RFP in high-copy	J107216	J107208	J107010

$H_{-3}dgLAC_{119,MC}P_{lac,HC}$	HSL-inducible dCas9 in low-copy plasmid with strong constitutive gPlac in medium-copy and the target P_{LlacO1} promoter driving RFP in high-copy	J107216	J107212	J107010
$H_{-3}dgTET_{100,LC}P_{lac,HC}$	Non-specific targeting control for the $H_{-3}dgLAC_{1XX,LC}P_{lac,HC}$ strains	J107218	-	J107010
$H_{-3}dgTET_{100,MC}P_{lac,MC}$	Non-specific targeting control for the $H_{-3}dgLAC_{1XX,MC}P_{lac,MC}$ strains	J107216	J107241	-
$H_{-3}dgTET_{100,MC}P_{lac,HC}$	Non-specific targeting control for the $H_{-3}dgLAC_{1XX,MC}P_{lac,HC}$ strains	J107216	J107214	J107010
Constructs with inducible sgRNA and constitutive dCas9				
$H_{-3}gTETd_{116}P_{tet,MC}$	HSL-inducible gPtet in low-copy plasmid with constitutive dCas9 in medium-copy and the target P_{LtetO1} promoter driving RFP in medium-copy	J107206	J107242	-
$H_{-3}gTETd_{116}P_{tet,HC}$	HSL-inducible gPtet in low-copy plasmid with constitutive dCas9 in medium-copy and the target P_{LtetO1} promoter driving RFP in high-copy	J107206	J107202	I13521
$H_{-3}gLACd_{116}P_{tet,MC}$	Non-specific targeting control for the $H_{-3}gTETd_{116}P_{tet,MC}$ strain	J107226	J107242	-
$H_{-3}gLACd_{116}P_{tet,HC}$	Non-specific targeting control for the $H_{-3}gTETd_{116}P_{tet,HC}$ strain	J107226	J107202	I13521
$H_{-3}gLACd_{116}P_{lac,MC}$	HSL-inducible gPlac in low-copy plasmid with constitutive dCas9 in medium-copy and the target P_{LlacO1} promoter driving RFP in medium-copy	J107226	J107243	-
$H_{-3}gLACd_{116}P_{lac,HC}$	HSL-inducible gPlac in low-copy plasmid with constitutive dCas9 in medium-copy and the target P_{LlacO1} promoter driving RFP in high-copy	J107226	J107202	J107010
$H_{-3}gTETd_{116}P_{lac,MC}$	Non-specific targeting control for the $H_{-3}gLACd_{116}P_{lac,MC}$ strain	J107206	J107243	-
$H_{-3}gTETd_{116}P_{lac,HC}$	Non-specific targeting control for the $H_{-3}gLACd_{116}P_{lac,HC}$ strain	J107206	J107202	J107010
$IgTETd_{116}P_{tet,MC}$	IPTG-inducible gPtet in low-copy plasmid with constitutive dCas9 in medium-copy and the target P_{LtetO1} promoter driving RFP in medium-copy	J107230	J107242	-
$IgTETd_{116}P_{tet,HC}$	IPTG-inducible gPtet in low-copy plasmid with constitutive dCas9 in medium-copy and the target P_{LtetO1} promoter driving RFP in high-copy	J107230	J107202	I13521
$IgLUXd_{116}P_{tet,MC}$	Non-specific targeting control for the $IgTETd_{116}P_{tet,MC}$ strain	J107229	J107242	-
$IgLUXd_{116}P_{tet,HC}$	Non-specific targeting control for the $IgTETd_{116}P_{tet,HC}$ strain	J107229	J107202	I13521
$IgLUXd_{116}P_{luxRep,MC}$	IPTG-inducible gPluxH in low-copy plasmid with constitutive dCas9 in medium-copy and the target P_{luxRep} promoter driving RFP in medium-copy	J107229	J107244[a]	-

IgLUXd$_{116}$P$_{luxRep,HC}$	IPTG-inducible gPluxH in low-copy plasmid with constitutive dCas9 in medium-copy and the target P$_{luxRep}$ promoter driving RFP in high-copy	J107229	J107202	J107100[b]
IgTETd$_{116}$P$_{luxRep,MC}$	Non-specific targeting control for the IgLUXd$_{116}$P$_{luxRep,MC}$ strain	J107230	J107244[a]	-
IgTETd$_{116}$P$_{luxRep,HC}$	Non-specific targeting control for the IgLUXd$_{116}$P$_{luxRep,HC}$ strain	J107230	J107202	J107100[b]
IgLUXd$_{116}$P$_{122,HC}$	IPTG-inducible gPluxH in low-copy plasmid with constitutive dCas9 in medium-copy and the target P$_{122}$ promoter driving RFP in high-copy	J107229	J107202	J107111[b]
IgLUXd$_{116}$P$_{2,HC}$	IPTG-inducible gPluxH in low-copy plasmid with constitutive dCas9 in medium-copy and the target P$_{2}$ promoter driving RFP in high-copy	J107229	J107202	J107101[b]
IgLUXd$_{116}$P$_{44,HC}$	IPTG-inducible gPluxH in low-copy plasmid with constitutive dCas9 in medium-copy and the target P$_{44}$ promoter driving RFP in high-copy	J107229	J107202	J107105[b]
IgLUX$_{d[n]}$d$_{116}$P$_{luxRep,MC}$	IPTG-inducible gPluxH variants with [n]-nt truncations in low-copy plasmid with constitutive dCas9 in medium-copy and the target P$_{luxRep}$ promoter driving RFP in medium-copy	J107229[c]	J107244[a]	-
IgLUX$_{e[n]}$d$_{116}$P$_{luxRep,MC}$	IPTG-inducible gPluxH variants with [n]-nt mismatching extension in low-copy plasmid with constitutive dCas9 in medium-copy and the target P$_{luxRep}$ promoter driving RFP in medium-copy	J107229[c]	J107244[a]	-
IgLUX$_{m[n]}$d$_{116}$P$_{luxRep,MC}$	IPTG-inducible gPluxH variants with mismatches (numbered and described in the manuscript) in low-copy plasmid with constitutive dCas9 in medium-copy and the target P$_{luxRep}$ promoter driving RFP in medium-copy	J107229[c]	J107244[a]	-
Transcriptional cascades				
X1T	HSL-inducible tetR-based NOT gate with RFP as output and constitutive GFP	J107245	-	-
X1TL	lux-tet-lac cascade with RFP as output and constitutive GFP	J107246	-	-
HgTETP$_{tet}$	HSL-inducible gPtet in low-copy plasmid with constitutive dCas9 in medium-copy and the target P$_{LtetO1}$ promoter driving RFP in low-copy	J107249	J107202	-
HgTET$_{d[n]}$P$_{tet}$	HSL-inducible gPtet variants with [n]-nt truncations in low-copy plasmid with constitutive dCas9 in medium-copy and the target P$_{LtetO1}$ promoter driving RFP in low-copy	J107249[c]	J107202	-

HgTET$_{DEG[n]}$P$_{tet}$	HSL-inducible gPtet variants with degenerate nucleotides in low-copy plasmid with constitutive dCas9 in medium-copy and the target P$_{LtetO1}$ promoter driving RFP in low-copy	J107249[c]	J107202	-
HgTET$_{DEG9}$P$_{tet}$	HSL-inducible optimized gPtet variant (tgtcaatctctatcgcggat) in low-copy plasmid with constitutive dCas9 in medium-copy and the target P$_{LtetO1}$ promoter driving RFP in low-copy	J107252	J107202	-
H$_{-3}$gTET$_{DEG9}$P$_{tet}$	HSL-inducible (P$_{lux-3A}$) optimized gPtet variant (tgtcaatctctatcgcggat) in low-copy plasmid with constitutive dCas9 in medium-copy and the target P$_{LtetO1}$ promoter driving RFP in low-copy	J107254	J107202	-
HgTET$_{DEG9}$P$_{tet}$LP$_{lac}$	HSL-inducible (P$_{lux-3A}$) low-burden transcriptional cascade including an optimized gPtet variant (tgtcaatctctatcgcggat) and a lacI-based logic inverter in low-copy plasmid with constitutive dCas9 in medium-copy and the target P$_{LtetO1}$ promoter driving RFP in low-copy	J107255	J107202	-
NOR gate				
NORgP$_{luxRep}$	IPTG- and HSL-driven NOR gate	J107256	J107202	J107100[b]
NORcontrol	Non-specific targeting control for the NORgP$_{luxRep}$ strain	J107257	J107202	J107100[b]

[a]Vector backbone is J107055, analogous to pSB3K3 but with a promoter-less RFP expression cassette between the *SpeI* and *PstI* sites.

[b]Vector backbone is J61002, analogous to pSB1A2 but with a promoter-less RFP expression cassette between the *SpeI* and *PstI* sites.

[c]Vector backbone is J107056, analogous to pSB4C5 but with a promoter-less RFP expression cassette between the *SpeI* and *PstI* sites.

Supplementary Table S2. List of model parameters.

Parameter	Units	Value	Reference
δ_{X1}	AU/min[1]	0.2	(Pasotti et al., 2017)
α_{X1}	AU/min	24.33	(Pasotti et al., 2017)
K_{X1}	nM	6.71	(Pasotti et al., 2017)
η_{X1}	-	1.19	(Pasotti et al., 2017)
δ_T	AU/min	0.21	(Pasotti et al., 2017)
α_T	AU/min	4.56	(Pasotti et al., 2017)
K_T	AU	6.92	(Pasotti et al., 2017)
η_T	-	2.57	(Pasotti et al., 2017)
δ_L	AU/min	0.22	(Pasotti et al., 2017)
α_L	AU/min	0.76	(Pasotti et al., 2017)
K_L	AU	34.92	(Pasotti et al., 2017)
η_L	-	1.93	(Pasotti et al., 2017)
J_{tet}	min/AU	0.31	(Pasotti et al., 2017)
$\Sigma_{X\lambda}$	-	0.36	(Pasotti et al., 2017)
γ_{tet}	min^{-1}	0.0173	(Pasotti et al., 2017)
γ_{lac}	min^{-1}	0.0533	(Pasotti et al., 2017)
a	min^{-1}	0.0167	(Pasotti et al., 2017)
μ	min^{-1}	0.006	This study
δ_{deg9}	AU/min	0.48	This study
α_{deg9}	AU/min	6.5	This study
K_{deg9}	AU	14.6	This study
η_{deg9}	-	1.11	This study
Σ_C	-	0.45	This study
δ_I	AU/min	0.04	This study
α_I	AU/min	4.77	This study
K_I	µM	2.94	This study
η_I	-	2.46	This study
K_C	AU	11.64	This study
U	-	676.3	This study
K_H	nM	2.2E+4	This study

[1]AU: arbitrary units of RFP fluorescence.
